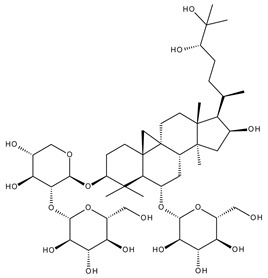# The Optimized Delivery of Triterpenes by Liposomal Nanoformulations: Overcoming the Challenges

**DOI:** 10.3390/ijms23031140

**Published:** 2022-01-20

**Authors:** Andreea Milan, Alexandra Mioc, Alexandra Prodea, Marius Mioc, Roxana Buzatu, Roxana Ghiulai, Roxana Racoviceanu, Florina Caruntu, Codruţa Şoica

**Affiliations:** 1 Faculty of Pharmacy, “Victor Babeş” University of Medicine and Pharmacy, 2 E. Murgu Sq., 300041 Timişoara, Romania; andreea.milan@umft.ro (A.M.); alexandra.petrus@umft.ro (A.M.); alexandra.ulici@umft.ro (A.P.); roxana.ghiulai@umft.ro (R.G.); babuta.roxana@umft.ro (R.R.); codrutasoica@umft.ro (C.Ş.); 2 Research Centre for Pharmaco-Toxicological Evaluation, “Victor Babes” University of Medicine and Pharmacy, Eftimie Murgu Sq., No. 2, 300041 Timişoara, Romania; 3 Faculty of Dental Medicine, “Victor Babeş” University of Medicine and Pharmacy Timişoara, 2 Eftimie Murgu Street, 300041 Timişoara, Romania; 4 Faculty of Medicine, “Victor Babeş” University of Medicine and Pharmacy Timişoara, 2 Eftimie Murgu Street, 300041 Timişoara, Romania; caruntu.florina@umft.ro

**Keywords:** liposomes, triterpenes, targeted delivery, nanocarriers, nano-therapy

## Abstract

The last decade has witnessed a sustained increase in the research development of modern-day chemo-therapeutics, especially for those used for high mortality rate pathologies. However, the therapeutic landscape is continuously changing as a result of the currently existing toxic side effects induced by a substantial range of drug classes. One growing research direction driven to mitigate such inconveniences has converged towards the study of natural molecules for their promising therapeutic potential. Triterpenes are one such class of compounds, intensively investigated for their therapeutic versatility. Although the pharmacological effects reported for several representatives of this class has come as a well-deserved encouragement, the pharmacokinetic profile of these molecules has turned out to be an unwelcomed disappointment. Nevertheless, the light at the end of the tunnel arrived with the development of nanotechnology, more specifically, the use of liposomes as drug delivery systems. Liposomes are easily synthesizable phospholipid-based vesicles, with highly tunable surfaces, that have the ability to transport both hydrophilic and lipophilic structures ensuring superior drug bioavailability at the action site as well as an increased selectivity. This study aims to report the results related to the development of different types of liposomes, used as targeted vectors for the delivery of various triterpenes of high pharmacological interest.

## 1. Introduction

The introduction of drugs derived from synthetic organic chemistry in the XXth century has marked the phenomenal advance of therapy in all medical fields due to their intense biologic effects and economic feasibility for large-scale preparation. However, a major disadvantage of synthetic drugs lies in their adverse effects such as the high, intolerable organ toxicity exhibited by anti-neoplastic drugs. Currently, this issue is mitigated by the development of modern and sophisticated therapies such as gene therapy, immunotherapy, or targeted therapy which show promising clinical outcomes but at high costs. A more approachable path consists of the replacement of synthetic drugs with natural compounds which exhibit similar pharmacological activities and can be chemically modified in order to obtain a suitable bioavailability [1]. Amongst all-natural bioactive compounds, triterpenes represent one of the most promising categories, not only for their large plethora of compounds but also for their wide range of pharmacological activities, such as anti-tumor, antioxidant, anti-viral, anti-microbial, cardioprotective, antidiabetic, neuroprotective, etc., [2]. Even though triterpenes exhibit such a significant pharmacological potential, their low solubility, hence bioavailability, leads to the need of finding proper formulations to enhance their biomedical activities [3].

Microtechnology and nanotechnology have revolutionized the 21st century of the pharmaceutical and biotechnological industry that are being viewed as powerful tools for basic research, imaging, and especially for enhanced drug delivery. The introduction of micro and nano-drug delivery systems provided the opportunity to obtain an improved therapeutic response with lower drug quantities while maintaining a high safety profile. The development of nanotechnology had a huge impact on creating new pharmaceutical products with significantly improved bioavailability [4], safety, and patient compliance, thus offering the possibility of delivering highly lipophilic or chemically unstable drugs [5]. The use of nanoparticles is not new to today’s technology; it originated in the medieval period when nano-sized gold particles were used in order to stain glass windows of churches, coloring them in orange, purple, red, and green according to their size [6].

Besides the clear distinction in particle size (1–100 nm for nanoparticles, 100–1000 nm for sub-microparticles, and 1–1000 μm microparticles), when comparing nanoparticles with microparticles as delivery systems, both possess properties that can make either of them more or less useful depending on the application type (chapter). Microparticles as well as microspheres and microcapsules, were developed for multiparticulate drug delivery system formulations. Due to their structural and functional abilities, they are considered to be suitable, tolerable, and convenient for drug administration via several routes. Despite their advantage to act locally, as microcarriers do not traverse into the interstitium and can consecutively incorporate toxic substances, when aiming for a systemic targeted delivery this is a shortcoming [7]. Consecutively, nanocarriers have evolved in order to overcome the disadvantage of microcarriers for systemic targeted delivery, as nanocarriers are able to cross through biological barriers [8].

Among various types of nanoparticles, lipidic vesicular systems, including liposomes, are nano-vehicles with many advantages due to their unique physical and chemical properties as well as high potency and ability to encapsulate a large number of different molecules [9]. Additionally, this type of nanoformulation exhibits high flexibility in preparation allowing it to undergo several types of surface modifications for extensive use in cutaneous applications, gene therapy, or as drug delivery systems in many pathologies such as cancer, HIV, tuberculosis, and brain pathologies [10].

This research aims to review the main methods used for triterpenoids inclusions in different types of liposomes while focusing on the positive biological results that could contribute to the future development of natural treatments. The literature data used to prepare the present review were identified by accessing internationally recognized databases such as PubMed, Web of Science, Science Direct, and Springer. Primary Medical Subject Headings [MeSH] terms such as “liposomes”, “triterpenes”, “pentacyclic triterpenes”, “drug delivery systems”, “nanotechnology” and regular keywords such as “vesicular systems’’, “ liposomal formulations”, ‘’betulinic acid liposomes’’, oleanolic acid liposomes’’, ‘’ursolic acid liposomes’’, lupeol liposones’’, ‘’boswellic acid liposomes’’, “glycyrrhetinic acid liposomes’’, “target therapy” combined with Boolean terms, were used to obtain the articles of interest. Only original articles and clinical trials published in English-language were included in this review. The reference list of the cited papers was examined in detail in order to extract other additional relevant literature data. No restrictions were applied regarding the publication date.

## 2. Nanotechnology and Nano-Therapy

In comparison to traditional therapeutics, which focus mostly on obtaining stable and tolerable therapies, nano-therapy aims for safer, more efficient, and less toxic treatment that can also increase patient compliance [11].

**Figure 1:**
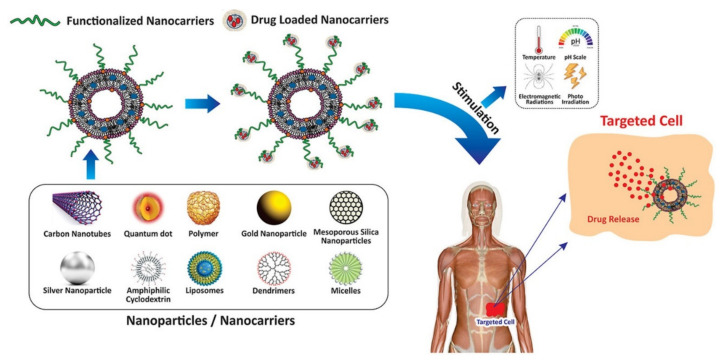
Representation of different nanoparticles reaching targeted sites. Reprinted from Journal of Drug Delivery Science and Technology, Vol 62, Afzal Shah, Saima Aftab, Jan Nisar, Muhammad Naeem Ashiq, Faoza Jan Iftikhar, Nanocarriers for targeted drug delivery, vol. 62, 102426, Copyright (2022), with permission from Elsevier. Reprinted from the Lancet, vol. 62, Afzal Shah, Saima Aftab, Jan Nisar, Muhammad Naeem Ashiq, Faoza Jan Iftikhar, Nanocarriers for targeted drug delivery, 102426, Copyright (2022), with permission from Elsevier [13].

Nanocarriers can be defined as nano-scaled formulations whose size may vary from 10 to 100 nm, which encapsulate and transport various drugs in order to achieve a targeted therapy, reaching high concentrations in specifically chosen tissues, with a reduced to zero general body toxicity (Figure 1) [12].

For the process of formulating nano-compounds, several characteristics need to be taken into consideration:

The nano-carrier must be properly formulated to easily access the biological action site and avoid any biochemical or enzymatic degradation that might occur during delivery [14].

Their chemical flexibility that manifests itself through changes in shape, size, and composition should be exploited in order to provide higher absorption in various tissues [15].

Nanoparticles must be thoroughly assessed in terms of size, morphology, and surface charge, parameters that might influence their in vivo distribution or potential toxicity; advanced microscopic techniques such as scanning electron microscopy (SEM), transmission electron microscopy (TEM), and atomic force microscopy (AFM) can be employed [16].

Chemical modifications can be conducted in order to increase their long-term stability and, subsequently, to improve their bioavailability [17].

The efficacy of nanocarriers depends on their size, shape, and morphology. Nanocarriers resolve many drawbacks that diminish the applicability of synthetic and natural compounds in therapy, such as in vivo instability, decreased bioavailability and solubility, low absorption, lack of target-specific delivery, and adverse effects. Nanoformulations significantly enhance the bioavailability of highly lipophilic drugs by improving their pharmacokinetic parameters and by reducing their interactions with intracellular proteins, while providing a targeted delivery to a specific site [18].

Besides the various advantages of nanoformulated drugs, several drawbacks need to be addressed. These include: (i) the difficulty in the manufacturing processes and potential stability issues which may appear during formulation, storing, and shipping; (ii) high pressure or temperature could severely cause damage to the formulated drug by causing changes in particles’ crystallinity; (iii) sedimentation, crystal growth, or agglomeration may occur during storing and shipping [19].

### 2.1. Classification of Nanocarriers

**Figure 2:**
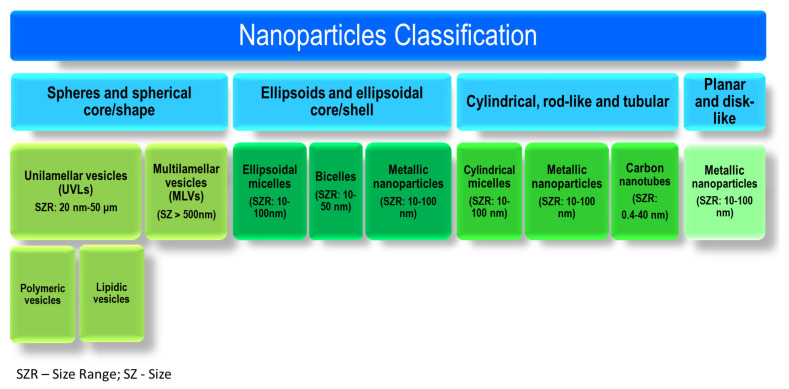
Classification of nanoparticles according to their morphology.

The properties of nanoparticles highly depend on their size and morphology, factors that have a significant influence on particles’ target efficiency [20]. According to their morphology, nanocarriers can be divided into four categories: (i) spheres and spherical core/shape-like nanoparticles; (ii) ellipsoids and ellipsoidal core/shell-like nanoparticles; (iii) cylindrical, rod-like and tubular nanoparticles; and (iv) planar and disk-like nanoparticles, which are all presented in Figure 2.

Depending on the transformation it undergoes in the organism, nanocarriers can also be classified into another two main categories: (i) disintegrative and (ii) non-disintegrative. The disintegrative nanoparticles, represented by organic nanoparticles such as polymeric nanoparticles and lipidic vesicular systems, undergo hydrolysis and degradation and release the active compound after reaching the targeted sites [21,22]. Contrarily, non-disintegrative nanoparticles, represented by inorganic nanoparticles such as metallic nanoparticles and quantum dots, are non-biodegradable; after delivering the active compound to the targeted site they are rapidly eliminated, due to their high toxicity, renally or hepatobiliary as such. Non-disintegrative nanoparticles possess a better therapeutic potential and are usually used in the field of imaging and photothermal therapy [23].

Nanotechnology is rapidly advancing in the field of personalized therapy; currently, various nanotools are under development and investigation due to their medical application in in vivo imaging and therapeutics [24]. Some main types of nanocarriers that have been further explored in the last decades for their medical applications in personalized therapy and imaging are presented in Table 1.

As presented above, nanocarriers provide a far superior potential compared to the conventional therapies [54]; this potential could be further harnessed by choosing routes of administration that are suitable for each nanocarrier, in order to obtain a better bioavailability and higher patient compliance. The classification of nanoformulations by their routes of administration with their respective advantages and drawbacks is presented in Table 2.

### 2.2. Biomedical Applications of Nanocarriers

One of the greatest challenges when developing nanocarriers loaded with various drugs for biomedical applications is ensuring a direct and efficient delivery. Consecutively, to preferentially deliver nanocarriers to diseased cells and tissues whilst maintaining minimal accumulation in healthy cells and tissues, various targeting strategies were developed:

Active targeting relies on a pair of nanocarriers loaded with a specific drug and ligand that specifically targets the selected cells or tissues [90].

Physical targeting is based on physical properties of the system nanocarrier–target tissue, by using pH-sensitive, temperature-sensitive, ultrasound-sensitive, and magnetic-sensitive systems [91].

Passive targeting relies on the accumulation of the drug-loaded nanoparticles in a diseased area through its leaky vasculature; usually in tumors and inflamed regions [92].

The ‘’stealth’’ effect refers directly to nanocarriers used for passive targeting whose shell is modified with polymers (polyethylene glycol-PEG, polyacrylamide, polyvinylpyrrolidone, polysaccharides, or dextrans), used to protect the nanocarriers from being degraded by the reticuloendothelial system (RES) [93].

**Figure 3:**
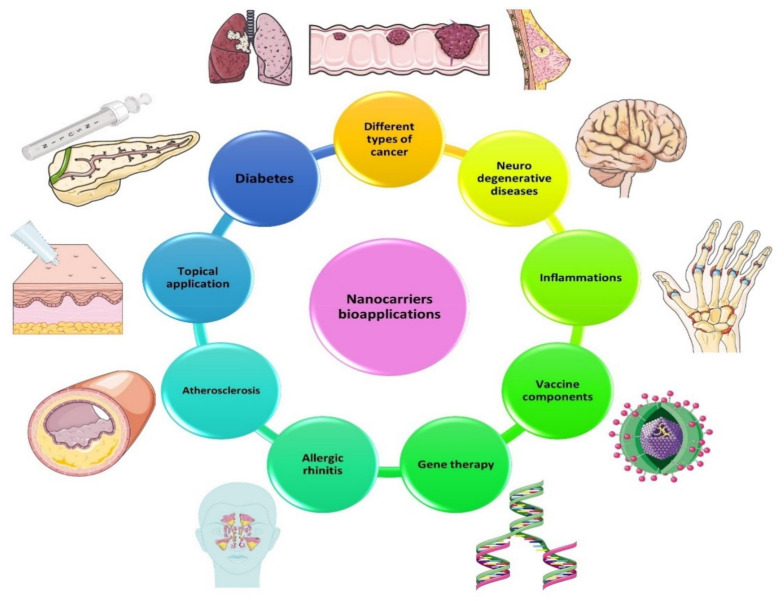
Different biomedical applications for nanocarriers.

Nanocarriers’ wide range of biomedical applications makes them prone to constant evaluation and improvement at an accelerated rate over the years. Despite their primary use as carriers of anti-neoplastic drugs, there are many other applications in diseases such as diabetes, neurodegenerative diseases (Alzheimer’s disease, Parkinson’s disease, Huntington’s disease), chronic inflammations, inflammatory diseases (inflammatory bowel disease), venous thromboembolism [94,95], and others that are summarized in Figure 3.

## 3. Lipidic Vesicular Systems

**Figure 4:**
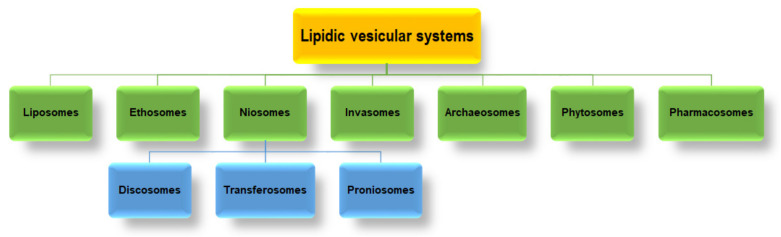
Examples of lipidic vesicular systems.

Lipid-based nanocarriers can also be classified into different vesicular systems, some of these are displayed in Figure 4.

### 3.1. Ethosomes

Ethosomes are lipid-based vesicular systems that are structurally very similar to liposomes but with a higher bioavailability and significant superior skin permeation proprieties [96]. These characteristics are offered by their high concentration of ethanol (20–45%) that acts as an efficient permeation enhancer by increasing the fluidity of the lipidic bilayers of the cellular membranes and by lowering the ethosome density [96]. Furthermore, it seems that ethosomes are deformable in many ways and can pass between skin corneocytes [97]. As a result, ethosomes can easily penetrate into the stratum corneum and skin barrier, otherwise a limiting step in the transdermal route [96,98].

**Figure 5:**
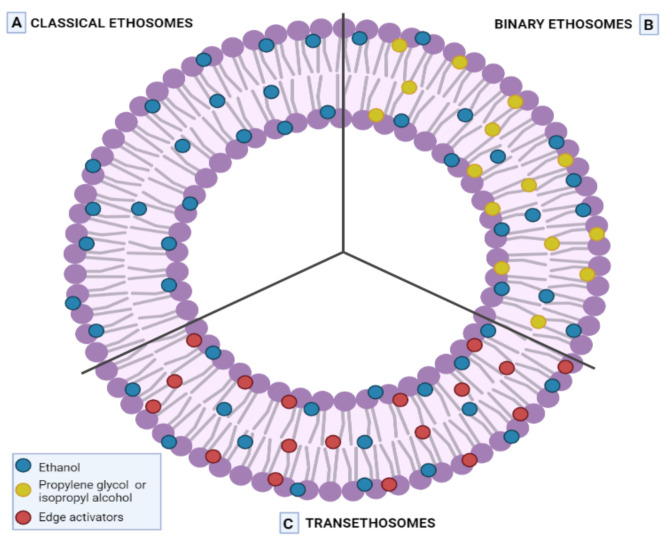
Structural representation of ethosomes: classical ethosomes (**A**), binary ethosomes (**B**), and transethosomes (**C**). Created with BioRender.com (accessed on 10 January 2022).

The pursuit to improve the properties of this type of carrier led researchers to the development of a new generation of ethosomes: binary ethosomes and transethosomes, depicted in Figure 5 [99].

Binary ethosomes are formulated not only with ethanol but also with propylene glycol (PEG) and isopropyl alcohol. The last two offer improved stability and permeation proprieties due to their higher hygroscopicity and viscosity. Moreover, this new type of binary ethosome is able to carry an increased amount of drugs into the deeper layers of the skin [100].

Transethosomes are lipid vesicles that were based on transfersomes and ethosomes. They contain phospholipids, high ethanol amounts (≅30%), and edge activators (Span 60, 65, 80; Tween 20, 60, 80; sodium deoxycholate, etc.) that offer, as compared to ethosomes, enhanced vesicle elasticity and increased skin permeation/penetration capacity [101,102].

### 3.2. Niosomes (Non-Ionic Surfactant- Based Vesicles)

**Figure 6:**
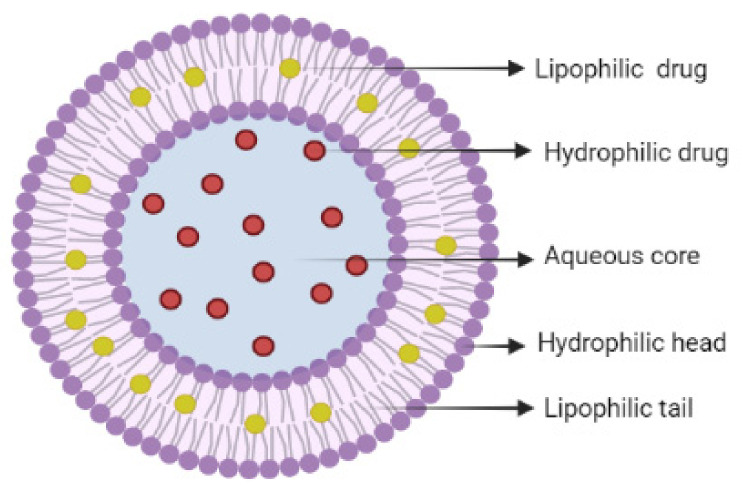
General structural representation of niosomes. Created with BioRender.com (accessed on 10 January 2022).

Niosomes are vesicular systems that are formed from a non-ionic surfactant and an aqueous core, that form bilayer structures due to their amphiphilic nature (Figure 6). Niosomes can be prepared by the same methods as liposomes and can be used in the cosmetic, pharmaceutical, and food science fields [103]. However, compared to liposomes, they possess several advantages including increased physical stability, lower production cost, and ease of storage [104]. Niosomes can be utilized as transdermal delivery systems on account of their superior skin penetration proprieties, for topical vaccine delivery, and ocular delivery due to their low toxicity [105,106].

Chen S et al. classified niosomes into different groups based on their composition and biomedical applications [107]:

(a) Elastic niosomes are composed of cholesterol, surfactants, water, and ethanol. They are flexible and can infiltrate into pores that are significantly smaller than their size, without altering their structure, and hence, are prone to be used in topical or transdermal drug delivery [108].
(b) Discomes are large vesicular thermosensitive systems (their structure changes with temperatures above 37 °C) that are generally used as ocular delivery systems [109].
(c) Transferosomes are highly deformable lipid vesicles constructed with an interior aqueous cavity that is encircled by a lipid bilayer that exhibits adaptive properties due to the presence of surfactants such as tween 80, Span 80, and sodium cholate in the vesicular membrane. These elastic properties ensure a rapid penetration through the skin of transferosomes loaded with considerable amounts of therapeutic agents [110,111].
(d) Aspasomes are prepared using cholesterol, a negatively charged lipid (diacetyl phosphate), and ascorbyl palmitate, which is more chemically stable than ascorbic acid and whose lipophilic nature improves skin penetration [112]. Moreover, due to the increased antioxidant potency of aspasomes they can be used as transdermal drug delivery systems in skin pathologies associated with increased reactive oxygen species production [112].
(e) Bola niosomes are made up of a new surfactant (bola: α,ω-hexadecylbis-(1-aza-18-crown-6), Span 80, and cholesterol which facilitates the formation of colloidal structures (~200 nm) that are capable to improve skin permeation of highly hydrophobic drugs [113]. Bola niosomes have proved their effectiveness in topical applications as carriers of hydrophilic anti-tumoral drugs [113].
(f) Proniosomes are dry, free-flowing formulations obtained by coating a layer of a non-ionic surfactant on a hydrophilic carrier that needs to be non-toxic and free-flowing, such as maltodextrin, sorbitol, mannitol, lactose, and glucose monohydrates [114]. Their stability is far superior as compared to noisome, while their pharmacological applications include not only transdermal deliveries but also pulmonary delivery for dry powder inhalers [115,116].

### 3.3. Exosomes

**Figure 7:**
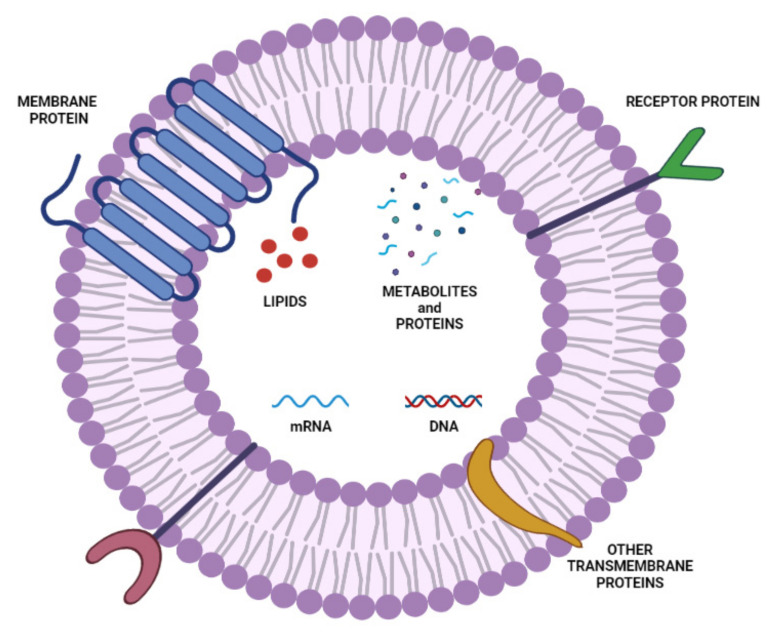
Exosome structure and content. Created with BioRender.com (accessed on 10 January 2022).

Exosomes are small-sized (30–120 nm) unilamellar vesicles that are shaped like a cup and are composed of a lipid bilayer, with a diameter of 40–100 nm, secreted by cells and that float on sucrose gradients, with a density varying from 1.13 up to 1.19 g/cm^3^ [117]. They contain a variety of proteins, nucleic acid, lipids, cytokines, and transcription factors (Figure 7). They play a role in cell-to-cell communication, participate in the regulation of physiological progress, and also in the immunologic and pathological processes of several diseases (viral diseases, cancer) [118]. Moreover, exosomes are secreted in all types of cells and released in body fluids (saliva, plasma, urine, and breast milk) [118]. Exosomes can be classified into natural and engineered exosomes, being able to deliver a variety of active compounds and are used not only for drug delivery, but also as biomarkers for early detection of cancer and treatment, and in different types of inflammations [119,120].

### 3.4. Invasomes

**Figure 8:**
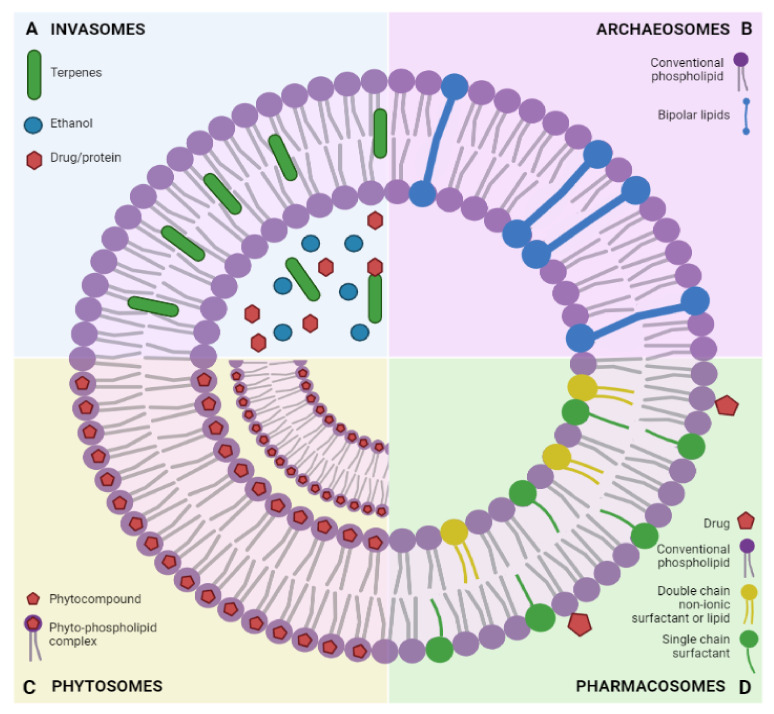
The structure and content of some types of lipidic vesicular systems: invasomes (**A**), archaeosomes (**B**), phytosomes (**C**), and pharmacosomes (**D**). Created with BioRender.com (accessed on 10 January 2022).

Invasomes are deformable lipidic vesicles prepared with phospholipids, ethanol, and terpenes (Figure 8A), components that can disrupt the lipid packing of the stratum corneum and thus, assuring an amplified skin penetration of drugs or proteins [121]. There are a plethora of biomedical applications using invasomes as nanocarriers; studies reported their use for the treatment of renal diseases [122], acne, hypertension, cancer, erectile dysfunction, and pustular folliculitis [123].

### 3.5. Archaeosomes

Archaeosomes are vesicular systems that are composed of natural or synthetic archaeobacterial ether lipids that present at sn-2,3 glycerol carbons branched phytanyl chains that are attached via ether bonds (Figure 8B) [124]. An increased stability was observed in archaeosomes with saturated phytanyl chains; these derivatives can be stored in methanol/chloroform for years at room temperature without suffering any chemical alterations [124]. Studies have demonstrated that archaeosomes are efficient vaccine delivery systems that present potent adjuvant activity, due to their propriety of induction humoral and cell-mediated immunity, in murine models with different types of cancers and intracellular infections [125,126].

### 3.6. Phytosomes

Phytosomes (phyto-phospholipid complexes) are vesicular systems that encapsulate polyphenolic constituents (Figure 8C) in order to enhance their low bioavailability, and thus are created structures that possess far superior skin permeation and penetration properties and even increased oral absorption [127]. Phytosomes are formed by complexing the active phytocompounds with phospholipids in different ratios; the phytocompounds are bound via hydrogen bonds to the polar moieties of the phospholipids [127]. Their biomedical applications are focused on topical treatments due to their high absorption capacity through the skin, impressive physical stability, and other improvements in skin function, such as increasing hydration, collagen structure, and enzyme balance [128].

### 3.7. Pharmacosomes

Pharmacosomes are amphiphilic vesicular systems of drugs that possess at least one active hydrogen atom, covalently bound to phospholipids in equimolar concentrations, thus providing a greater drug load (Figure 8D) [129]. Their amphiphilic nature offers the advantage of increased absorption through tissues and dissolution in gastrointestinal fluid [129]. Compared to conventional liposomes, they provide a superior targeted delivery by significantly increasing the bioavailability of several lipophilic drugs, providing greater entrapment results by ensuring no drug leakage due to their covalent bonds [130].

## 4. Liposomes as Targeted Delivery Systems

Liposomes were discovered by Alec D. Bangham in 1960 at the Braham Institute at Cambridge University and are mainly made up of one or more lipid layers that encapsulate a hydrophilic layer [131]. They possess a cylindrical shape and a diameter ranging from nanometres to several hundred micrometres; liposomes with a diameter between 50–450 nm are used in therapy [131]. It is considered that particles with a size of up to 100 nm can be classified as nanoparticles, whereas particles with a size ranging from 100 to 1000 nm can be classified as sub-microparticles [132].

**Figure 9:**
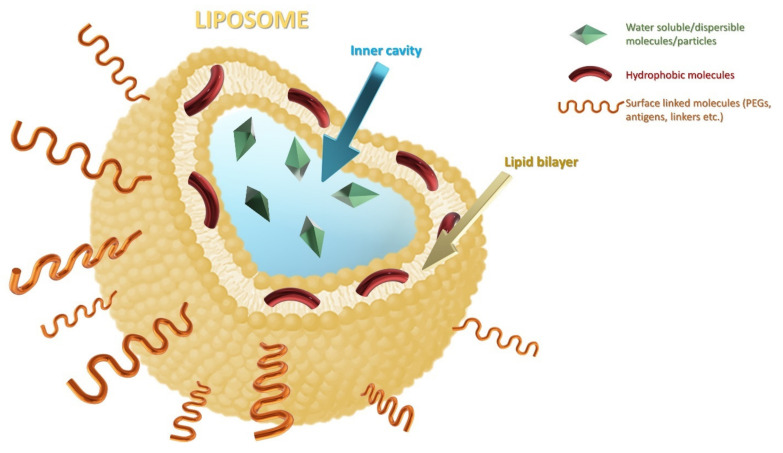
General schematic representation of a liposome structure.

This complex structure of liposomes is stable due to the interactions between the phospholipids which are composed of a hydrophilic head and two hydrophobic tails. On account of their amphiphilic characteristics, liposomes can entrap both hydrophilic and lipophilic drugs (Figure 9) [133].

The hydrophilic heads tend to interact with the aqueous environment due to the hydrogen bonds formed between them and the water molecules of the environment, while the hydrophobic chains aim to interact with each other due to the hydrophobic interactions that form the lipid bilayers and van der Waals forces that hold the tails together [134]. The lipid bilayers hydration can form: (i) multilamellar vesicles (MLVs), which are composed of concentric bilayers separated by hydrophilic portions, and (ii) unilamellar vehicles (UVs) that can be further classified into three distinct categories: giant unilamellar vehicles (GUVs: 10–100 μm), large unilamellar vehicles (LUVs: 100–500 nm), and small unilamellar vehicles (SUVs: 30–50 nm) [135].

Liposomes are one of the most developed types of nanoformulations, being the vehicle of choice due to their chemical versatility, high potency, increased capacity to encapsulate a significant amount of the active compound, and due to their structural resemblance to human cells that provides the opportunity to encapsulate DNA, proteins, and antibodies [136].

Examples of liposomal formulations with various drugs that are used in therapy or are still in clinical trials are listed in Table 3 and Table 4.

### 4.1. Advantages and Disadvantages of Liposomes

There are several advantages of this type of nanocarriers that made them be the nano-vehicle of choice:

(i) they provide high solubility to the lipophilic drugs that they encapsulate, that usually possess a low solubility; therefore a low bioavailability [184];
(ii) they can both entrap hydrophilic and lipophilic drugs, and release the drug at specific targets; moreover, their chemical versatility offers them the possibility to be modified to obtain a better selectivity and reduce their degradation during administration and storing [185];
(iii) they significantly reduce the acute toxicity of some highly toxic drugs and the exposure of sensitive tissues to those drugs; improving the drug’s therapeutic index and also their compatibility with the natural compounds that are mixed with, reducing the toxicity of the nanocarrier [186,187].

Nevertheless, despite their various benefits, there are some drawbacks that need to be taken into account; some of them have been overcome, such as drug leakage that was solved by polymerization of the lipids, while some of them are still hard to overcome, such as the high cost and the complexity of production [188].

### 4.2. Classification of Liposomes

**Figure 10:**
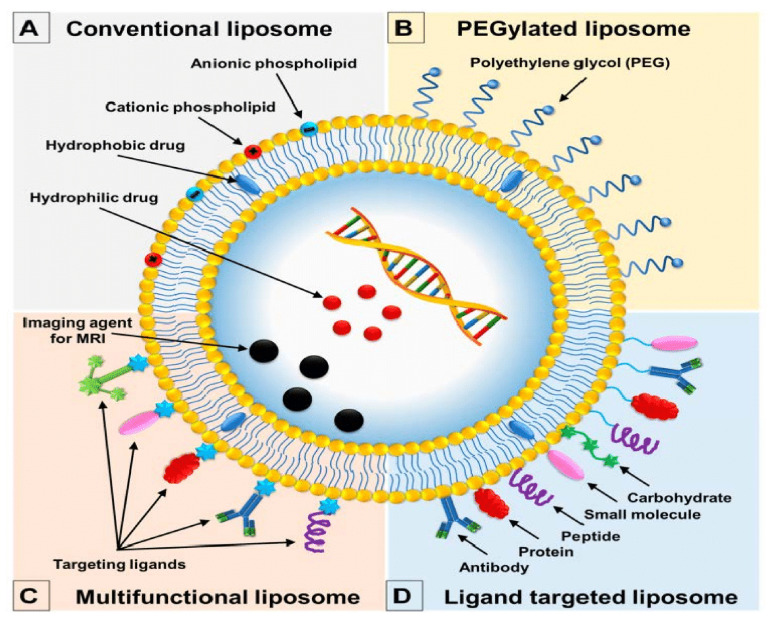
Classification of liposomes by surface modification strategies: conventional liposomes (**A**), PEGylated liposomes (**B**), multifunctional liposomes (**C**), and ligand-targeted liposomes (**D**). Reprinted with permission from [192]; copyright (2022). International Journal of Applied Pharmaceutics.

Liposomal drug delivery systems can be classified based on some individual criteria: size (small with a diameter of 30–70 nm, intermediate or large with a diameter >100 μm in size), lamellarity (unilamellar, oligolamellar, and multilamellar), method of preparation (obtained by reverse-phase evaporation vesicles, by the extraction method, and by dehydration-rehydration method), surface modification strategies (conventional liposomes, PEGylated liposomes, multifunctional liposomes, and ligand targeted liposomes—Figure 10) and mechanism of intercellular delivery (conventional liposomes, pH-sensitive liposomes, cationic liposomes, immuno-liposomes, long-circulating liposomes, and thermo-sensitive liposomes) [189,190,191].

#### 4.2.1. Unilamellar and Multilamellar Liposomes

**Figure 11:**
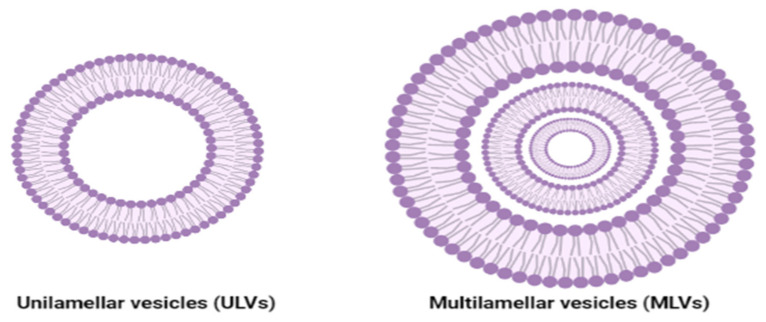
Structural representation of unilamellar and multilamellar vesicles. Created with BioRender.com (accessed on 10 January 2022).

Unilamellar vesicles (ULVs) or multilamellar vesicles (MLVs) can be obtained by choosing a certain method of preparation and synthesis such as the Bangham method for MLVs and ISCRPE and the SCRPE method for ULVs [191]. When comparing the properties of the two types of liposomes, it seems that UVLs (50–250 nm), which consist of only one lipid bilayer, tend to incorporate hydrophilic drugs since they possess a large aqueous interior, while MLVs (1–5 μm), that contain two or more lipidic bilayers oriented concentrically, are more suitable for lipophilic drugs (Figure 11) [191].

#### 4.2.2. Conventional Liposomes

Liposomal formulations have been explored widely for at least two decades due to their plethora of advantages and mostly for their possibility of incorporating various types of drugs in very small or very high quantities and delivering them on targeted sites that are otherwise hard to reach [189]. The most inconvenient drawback that it is encountered while using conventional liposomes is the fact that they are easily recognized and captured by the mononuclear phagocyte system (MPS) and cleared from blood circulation; moreover, as a result of their chemical composition, they are very unstable in the plasma because they interact with lipoproteins, thus they release the drug load into the plasma [193].

#### 4.2.3. Temperature-Sensitive Liposomes (TSLs)

**Figure 12:**
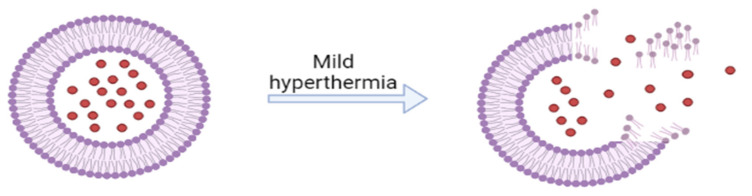
Schematic representation of temperature-sensitive liposomes. Created with BioRender.com (accessed on 10 January 2022).

TSLs are made up of temperature-sensitive lipids that change their microarchitecture from a solid and compact structure of gel to a loose structure of a liquid-crystal state; providing the property of releasing the drug at a temperature in which the lipids suffer a phase change—temperatures usually combine with local hyperthermia (40–45 °C), which is clinically established as a suitable treatment for solid tumors (Figure 12) [194]. TSLs are composed of dipalmitoyl phosphatidylcholine (which has a transition temperature = 41 °C) and might suffer modification for an increased drug release, better chemical stability (they might incorporate polyethylene glycol on TSLs), or for the adjustment of the temperature in which the TSLs release the payload (cholesterol, hydrogenated soy phosphatidylcholine-HSPC and 1,2-distearoyl-sn-glycero-3-phosphocholine might be incorporated) [195].

#### 4.2.4. pH-Sensitive Liposomes (PSLs)

Both TSLs and PSLs were designed as targeted delivery systems for their property to encapsulate the drugs and only release them when the body conditions are suitable for delivery, in a certain temperature range and pH range, accordingly [196].

**Figure 13:**
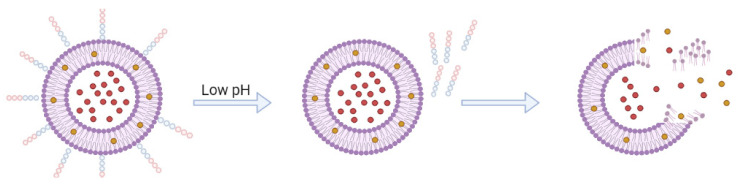
Schematic representation of pH-sensitive liposomes. Created with BioRender.com (accessed on 10 January 2022).

Commonly, PSLs are stable at a physiological pH, but at slightly acidic values of pH, usually in case of inflammations and infections, they are released when the lipidic bilayers are destabilized (Figure 13); being the most promising types of carriers for delivering genes, antisense oligonucleotides, and proteins [197]. The most used compound for the formulation of PSLs is phosphatidylethanolamine (PE) or its derivatives that contain an acidic moiety which is a stabilizer at a neutral pH; however, similar to conventional liposomes, PSLs are degraded by the MPS, lowering their pharmacological activity [198]. To address this issue many types of formulations have been tested; it was noticed that the combination of PE and lipids that possess a high transition temperature (such as cholesterol, HSPC, distearoylphosphatidylcholine) reduces drug leakage before being released at the targeted site [199].

#### 4.2.5. Ligand-Conjugated Liposomes

There are several types of molecules used as ligands (sugars, peptides, hormones, antibodies, proteins, enzymes), that are conjugated onto liposomal surfaces, to recognize the complementary molecules of the targeted cell [200]. Sugar-conjugated liposomes exploit the fact that malignant cells, by having an accelerated metabolism, require a much higher amount of glucose and therefore overexpress the glucose transporter (GLUT) on the cell membrane, thus providing glyco-conjugation to liposomes, representing a good targeting option for cancer therapy [201].

Proteins and polypeptides are suitable candidates for ligand conjugation due to their high density and chemical functionality that make them easy to modify, in order to improve their pharmacokinetics, pharmacological effect, and efficacy on binding [202]. By working on the premise that different types of tumor tissues overexpress the fibroblast growth factor (FGF) and FGF receptors, Cai et al. developed FGF-mediated cationic liposomes that delivered anti-cancer drugs at the tumor site, thus reducing its growth [203]. The delivery system was formulated by polymerizing the liposome with cholesterol and PEG that had FGF fragments chemically linked on its surface [203]. Considering the fact that a plethora of cancer tissues overexpress folate receptors, another promising ligand for targeted therapy is represented by forming folate-conjugated liposomes or preformulated liposomes that are linked covalently to folate polymers, using a post-insertion method [204].

Enzymes might also be attached to liposomes for controlling enzymatic activities; hydrophilic enzymes are encapsulated directly into the internal phase of the liposomes, providing them with stability even at high temperatures, or they can also be conjugated covalently on the surface of the liposomal membrane [205].

#### 4.2.6. Antibody-Targeted Liposomes (Immunoliposomes)

**Figure 14:**
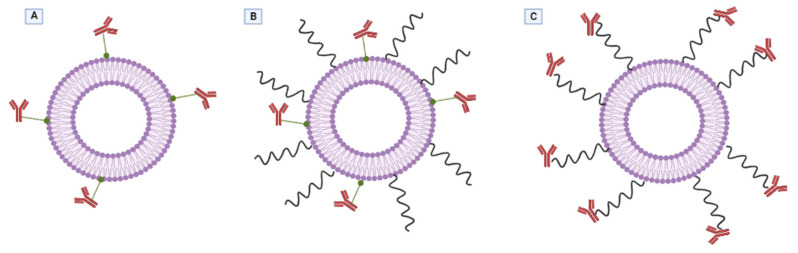
Schematic representation of antibody attachment to liposomes: antibodies are attached directly onto the surface of conventional liposomes (**A**), antibodies are attached directly onto the surface of PEGylated liposomes (**B**), and antibodies are attached to the end of PEG chains of liposomes (**C**). Created with BioRender.com (accessed on 10 January 2022).

Immunoliposomes are a special subcategory of ligand-conjugated liposomes that actively target various types of tumorous tissues using specific antibodies attached to liposomes that are recognized by specific receptors. Since 1980, there have been many early attempts of attaching the antibodies directly to the surface of conventional liposomes; however, recent studies improved their formulation by binding them on PEGylated liposomes, to raise their half-life and avoid a rapid clearance [206]. One major drawback of the PEGylation process was the limited availability of the ligand that occurred as a result of the antibody being hidden in the polymeric cover. Nowadays this disadvantage has been overcome; the antibodies are currently attached to the end of PEG chains to avoid inefficient binding and guarantee target recognition (Figure 14) [207].

#### 4.2.7. Sterically Stabilized (Stealth) Liposomes

**Figure 15:**
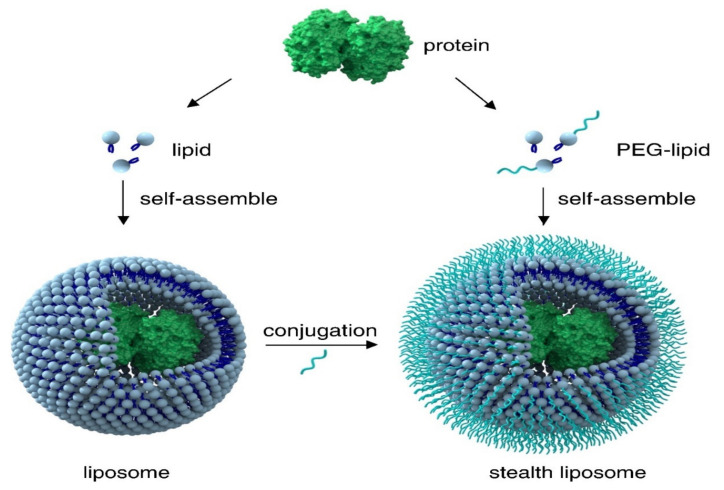
Formulation mechanism of stealth liposomes. Reprinted with permission from [212]; copyright (2022). Advanced Drug Delivery Reviews.

Stealth liposomes were designed to prevail over conventional liposomes’ drawback of predominant accumulation of the drug in organs and drug leakage before being delivered to the targeted sites while being suitable for all types of administration routes [208]. Therefore, the surface of the liposomes was modified by adding natural polymers. PEG is considered to be the first choice due to its versatility and high hydrophilia that leads to less interaction with plasmatic proteins. Moreover, PEG provides improved stability due to its property of being invisible to macrophages and thus, it can evade the immune system (mononuclear phagocyte system—MPS) and penetrate the blood–brain barrier, without producing any inflammatory response [209]. The main technique for creating stealth liposomes is the inclusion of PEG conjugates into the lipidic film of the liposome, while being hydrated, liposomes are formed with PEG polymers oriented onto the surfaces. While other techniques rely on attaching the PEG polymers to the pre-formed liposomes, the post-conjugation method relies on the covalent attachment of the polymeric component onto the liposome obtained previously [210]. Another technique for creating stealth liposomes is the post-insertion method, based on the incubation of liposomes with PEG–lipid conjugates in a hydrophilic solution [210]. Other polymers that can be used for creating stealth liposomes are: (i) polyacrylamide—formed from the polymerization of acrylamide, possessing a high hydrophilia; (ii) poly (2-methyl-2-oxazoline), (iii) poly (2-oxazoline)—preferred for its long-circulation properties and ability to not be detected by the immune system; (iv) poly (amino) acids—formed from repeating amino-acid units; (v) polyglycerol—possesses a structure similar to PEG; (vi) poly (vinylpyrrolidone)—is highly water soluble and a good formulation agent; (vii) dextran—a branched polysaccharide made of glucose units; (viii) polysorbates—esters formed from oily liquids formed from a derivatized sorbitan and fatty acids [211]. The construction principle for stealth liposomes is represented in Figure 15.

#### 4.2.8. Magnetoliposomes (MLs)

MLs were developed for a more accurate targeting delivery than conventional liposomes, usually being used as contrast agents for MRI and as controllers of cell signaling, tracking, and sorting [213]. The magnetic proprieties increase with the particle’s growth and subsequently, their effectiveness in biomedical applications. The only drawback of this type of liposome is the size of the magnetic core; while larger particles are more efficient, they are also harder to be incorporated into the liposome internal cavity [214]. Another promising use of MLs is in tumor treatment where, by using superparamagnetic hyperthermia, they can be better absorbed at the tumor level. However, the major drawback of this application is the necessity for high magnetic fields [215].

## 5. Liposomal Formulations of Triterpenoids Used in Drug Delivery

Terpenoids may be considered one of the most abundant and complex classes of natural compounds that exhibit such a wide range of pharmacological properties [216]. They are mainly extracted from volatile oils of several categories of medicinal plant groups: *Ranunculaceae*, *Araliaceae, Oleaceae, Labiatae, Pinaceae, Lauraceae, Rutaceae, Taxaceae, Magnoliaceae*, etc. [217]. They are formed by squalene cyclization in various species of medicinal herbs and can be classified by their number of isoprene units [218]; the subclass of triterpenes can be also subdivided into pentacyclic triterpenes and tetracyclic triterpenes according to their chemical structure [219]. The main representants of the pentacyclic triterpenes group are lupane, oleanane, and ursane derivatives, exhibiting antiviral, antineoplastic, anti-inflammatory, and antioxidant activities, while dammarane, lanostane, and cycloartane belonging to the tetraterpenic group (Table 5) present mostly cytotoxic and anti-neoplastic activities [220,221].

A well-known problem of triterpenes is their low bioavailability caused mainly by their high lipophilia and water insolubility, which significantly reduces gastrointestinal absorption of the compounds [222].

Researchers have focused on discovering methods for enhancing the bioavailability of triterpenoids. This review aims to summarize some methods obtained by the inclusion of these compounds in liposomes to acquire an improved effect and a targeted delivery.

### 5.1. Tetracyclic Triterpenes Liposomal Formulations

Cucurbitacin E (Cuc E) is an acylated compound belonging to the group of tetracyclic triterpenes and possessing anti-proliferative activity against various types of cancers. Habib et al. designed an experimental study in order to assess the potential use of liposomes as cell membrane models for the evaluation of the Cuc E effect on the membrane’s biophysical properties; the study clarified its ability to interact with lipid membranes and to alter domain formation; therefore, establishing its potential to be included in therapy as an anti-cancer agent. The liposomes were prepared using the reverse-phased evaporation method; the results showed the liposomal incorporation of Cuc E without altering the zeta potential and without inducing a phase separation, an aspect that could be very helpful for future liposomal formulations. In addition, it was established that CuC E interacted with the surface of the lipid vesicles and induced significant changes to the liposomes in terms of height and size thus leading, by extrapolation, to the conclusion that the phytocompound alters the lipid membrane structures [223].

### 5.2. Pentacyclic Triterpenes Liposomal Formulations

#### 5.2.1. Betulinic Acid

**Figure 16:**
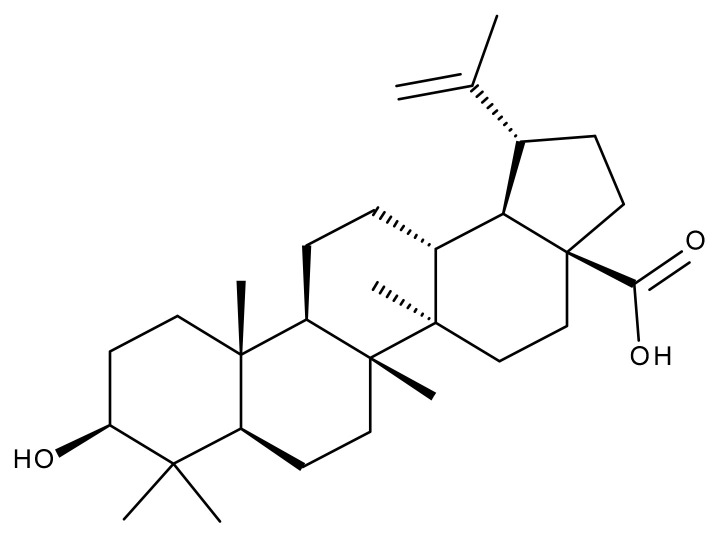
Chemical structure of betulinic acid.

**Figure 17:**
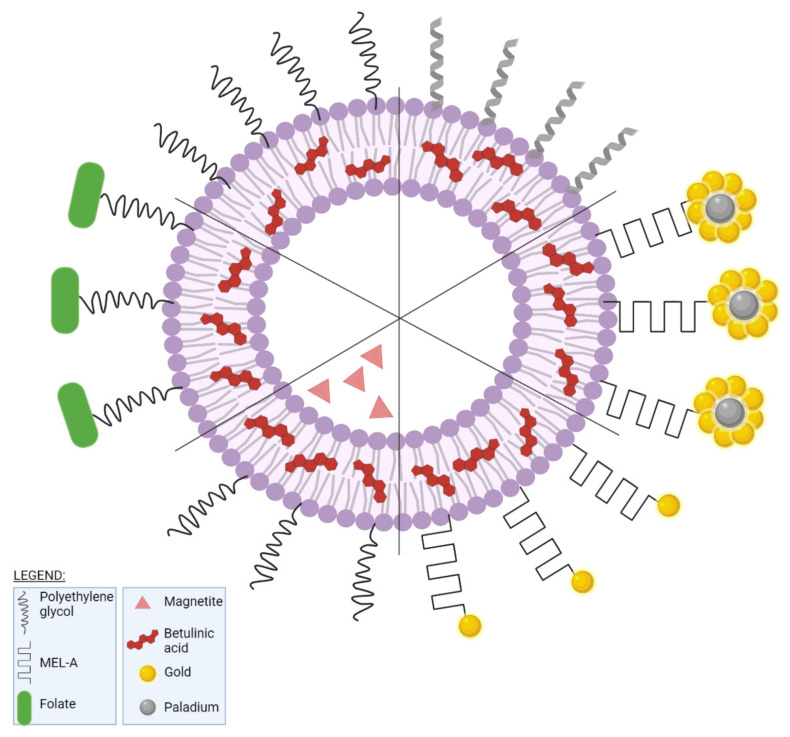
Schematic representation of different reported BA liposomal formulations. Created with BioRender.com (accessed on 10 January 2022).

Betulinic (BA) acid is a pentacyclic triterpene with a lupane structure (Figure 16), widely spread in nature with higher amounts being found in white birch tree (*Betula* spp.), jujube tree (*Ziziphus mauritiana* Lam.), eucalyptus bark (*Eucalyptus globulus* Labill.), etc. [224,225]; it exhibits a large range of pharmacological bioactivities consisting of anti-HIV, anti-malarial, anthelmintic, anti-depression, anti-hyperlipidemic [226,227] and anti-cancer activity on several distinct cancer cells such as ovarian, prostate, breast, brain, and Ewing’s sarcoma [228]. The various BA formulations discussed in this section are schematically represented in Figure 17.

In a study conducted by Liu Y et al., the bioavailability of BA was improved by its encapsulation in PEG-ylated liposomes; this type of liposome was selected due to its longer blood-circulating time compared to conventional liposomes as well as its low interactions with plasmatic proteins. BA liposomes containing soya lecithin, cholesterol, Tween-80, and PEG-2000 were obtained following the ethanol injection technique. The BA:lecithin mass ratio was optimized to reach a final formulation with a maximum BA encapsulation efficiency (95%). Due to its hydrophobic nature, BA was entrapped in the lipid bilayers of the liposomes, while the hydrophilic PEG chains formed an exterior layer that enfolded the liposomal surface. The resulting liposomes exhibited a mean diameter of 142 nm which seems to be the optimal size range (100–200 nm) for the accumulation in tumor tissues. In vitro and in vivo tests revealed a prolonged drug release of the phytocompound compared to conventional liposomes as well as a higher anti-tumor effect; therefore, the PEGylated BA liposomes may provide a more efficient alternative for cancer therapy in the future [229].

Jin et al. developed an experimental treatment for lung cancer using a cocktail of betulinic acid, parthenolide, honokiol, and ginsenoside Rh2 incorporated in PEG-ylated liposomes [230]. The liposomes were prepared by direct hydration of a lipidic film containing phosphatidylcholine:cholesterol:DSPE-PEG2000 (15:1:4) and were later tested in vitro using the cytotoxicity assay on the A549 cell line and in vivo on nude mice xenografted with A549 cancer cells. The determined encapsulation efficiency in respect to BA alone was 89.5% while cellular uptake of these liposomes was time dependent showing a maximum around 4 h incubation but also a slight decrease after 8 h incubation. The experiment showed that all phytocompounds exerted anti-proliferative effects and inhibited tumor growth. Moreover, the cocktail revealed a synergistic inhibitory effect between its components; the liposomal nanoformulation exhibited far superior anti-tumor effects to those recorded in the cisplatin group used as a positive control. Furthermore, the in vivo results were promising not only for the overall anti-cancer effect but also for the safety profile of the cocktail; the phytocompounds’ mixture did not induce any damage to major organs as opposed to the cisplatin group which exhibited kidney damage thus proving to be a valid option for future treatments of lung cancer [230].

Folate-functionalized PEG-ylated liposomes containing BA were designed and prepared by Guo et al. using the thin lipid film method. For this purpose, the authors used a modified lipid (folate–(NH_2_-PEG-NH^2^)–cholesterol), enzymatically linked through amidic bonds. The formulation folate group was located at the outer extremity of PEG, away from the lipidic bilayer. The obtained liposomes achieved an average size of 222 nm and showed high storage stability and no leakage after 3 months. Determined encapsulation efficiency was around 90%, similar to other BA PEG-ylated liposomes described above. The cytotoxic and targeting effects of these liposomes were examined on the positive folate receptor (FR) HepG2 and negative FR A549 cell lines, using the MTT assay. The results showed that the FR-targeted liposomes expressed higher cytotoxicity against FR(+) but not the FR(−) cancer cells thus revealing selective anti-tumor effects against HepG2 cells. In addition, the folate-decorated liposomes showed increased cytotoxic activity and higher uptake in HepG2 cells compared to conventional nontargeted liposomes; therefore, qualifying as potential drug carriers for the active targeting of tumor cells [231].

A new multifunctional nanoformulation was designed in order to be used in chemo- and photothermal therapy consisting of gold-nanoshell-based BA liposomes (AuNPs-BA-Lips). For this purpose, BA was entrapped in cholesterol-lecithin-based liposomes while glutathione was used as a bridge in order to create an Au–S bond which allowed the encapsulation of liposomes into gold nanoshells. Reported encapsulation efficiency (80.6%) was lower as compared to previously mentioned BA–PEG liposomes. The cellular uptake was determined in HeLa cells and evaluated quantitatively using fluorescence microscopy; the NIR light-excited liposomes exhibited significantly higher cellular uptake compared to non-irradiated liposomes due to the red-shifted absorption wavelength towards the NIR region. The anti-tumor effect combined with photothermal therapy was tested in vitro against 143B and HeLa cells, respectively, using the MTT assay method; the smart nanocarrier was able to rapidly converse the NIR light into heat which in turn triggered drug release, thus inhibiting cell growth by the combined effects of chemotherapy and hyperthermia. Furthermore, in vivo studies were performed on tumor-bearing Kunming mice, demonstrating the chemo and photothermal effect of AuNPs-BA-Lips; the combination of chemo- and photothermal therapy successfully penetrated tumor cells where the on-demand drug release triggered by NIR irradiation significantly increased its anti-tumor activity. Additionally, the induced hyperthermia acted via a double mechanism, simultaneously ablating tumor cells and increasing their permeability thus resulting in higher drug cellular uptake and an optimized overall anti-cancer effect [232]. The same principle was used by Liu et al., who designed new poly-branched Au–Pd bimetallic nanoflowers-coated BA liposomes (BA–Lips–Pd–Au NFs) in order to exploit the synergistic anti-cancer effects induced by the combination of chemo- and photothermal therapy; the bimetallic nanostructure showed strong photothermal conversion properties while BA was identified as an effective anti-tumor agent. Their cytotoxic effect was tested in vitro against HeLa cells by using the MTT assay and in vivo on U14 tumor-bearing mice; both in vitro and in vivo studies showed an increased inhibitory activity in cancer cells exerted by the combination therapy which proved significantly more effective than either therapy alone. The anti-tumor effect was also revealed as synergistic due to the hyperthermia which enhanced the cellular uptake of BA and also triggered its release thus subsequently increasing its cytotoxic activity; in addition, hyperthermia induced the ablation of cancer cells. Equally important, the novel multifunctional platform revealed high biocompatibility which promotes it as a future candidate for bimodal chemo-photothermal therapy [233].

Glycolipid biosurfactants such as mannosylerythritol lipid A (MEL-A) have the remarkable potential to stabilize liposomes and to increase their drug-carrying ability; a novel BA soy phosphatidylcholine-cholesterol liposome modified with biosurfactant was developed and tested on HepG2 cells. The liposomes were obtained through the lipid film hydration method using various mass ratios of cholesterol, soy phosphatidylcholine, and MEL-A, to reach a final optimal formulation regarding zeta potential, PDI, and encapsulation efficiency. The MEL-A modification affected the particle size and zeta potential, the resulting liposomes showing improved stability, although the encapsulation efficiency was not significantly changed showing a slight 1.77% decrease; moreover, MEL-A increased the transfection efficacy and accelerated the liposome’s entry into the tumor cells. Therefore, the authors concluded that the biosurfactant promoted the delivery of BA-loaded liposomes to HepG2 cells which, together with its intrinsic anti-tumor activity led to a synergistic anti-cancer effect [234].

Mullauer et al. conducted an in vivo study on A549 (lung cancer) and SW480 (colon cancer) xenograft models on athymic nude Foxn1 mice using BA formulated as small and large liposomes. The liposomes were synthesized by the film hydration method using a mixture of egg-phosphatidylcholine and egg-phosphatidylglycerol in a 10:2 molar ratio and various ratios of BA that later enabled to afford small (0.1–0.2 μm) and large liposomes (1–1.5 μm). Although the small liposomes exhibited the maximal drug incorporation (up to 1 mg/mL BA) while also preferentially targeting the tumor in a passive manner, when tested in vivo they failed to inhibit tumor growth presumably due to the overall small drug amount. Therefore, large liposomes were designed and prepared without the use of cholesterol, resulting in the incorporation of a drug amount six times higher compared to small liposomes (6 mg/mL BA); the presence of the incorporated BA produced a significant increase in the liposomes’ in vitro stability. In vivo tests showed a reduction in lung and colon tumor growth by more than 50% compared to the control group as a result of the parenteral/oral administration of BA-loaded large liposomes; the oral route proved to be less efficient compared to the i.v. one presumably due to the digestive process which hampers BA absorption. The authors concluded that, instead of functioning as targeted drug carriers in a similar manner with small liposomes, the large vesicles acted as solubilizing vehicles for the active phytocompound; the BA formulation significantly reduced colon and lung tumors and also succeeded in extending mice survival while completely lacking systemic toxicity [235].

Farcas et al. developed a new targeted delivery method for BA using magnetoliposomes as nanocarriers against breast cancer. A liposomal platform capable of incorporating both BA and magnetic iron oxide nanoparticles (MIONPs) was created in order to release the BA payload under hyperthermic conditions. These PEG-ylated liposomes were also obtained by the film hydration method where the lipid film containing the active BA was hydrated with a magnetite nanosuspension. The BA-MIONPs showed suitable diameters (under 200 nm, with a mean of 198 nm) for their biological purposes. The BA MIONPs were tested in vitro against MCF7 and MDA-MB-231 cell lines and were later compared to BA alone and BA liposomes under normothermic conditions. The BA-loaded magnetoliposomes exhibited a biocompatible phase transition temperature, superparamagnetic properties, and heating ability; the induced hyperthermia enhanced the anti-tumor effect of BA-loaded liposomes while selectively targeting breast cancer cells [236].

#### 5.2.2. Oleanolic Acid

**Figure 18:**
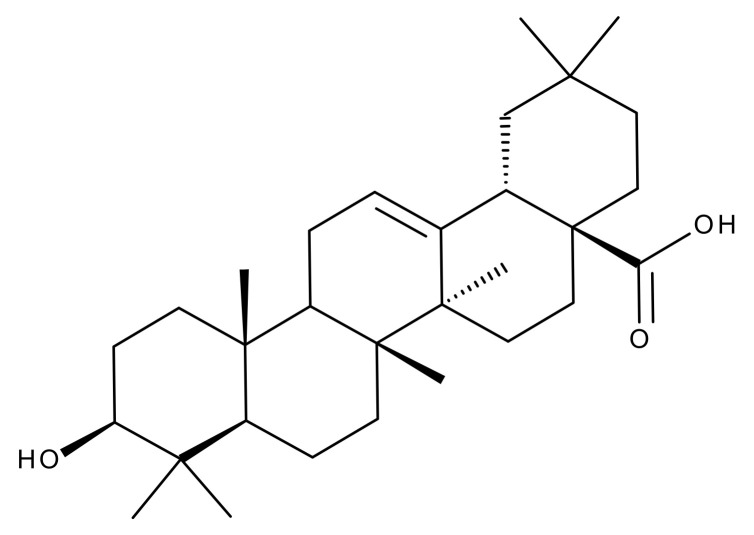
Chemical structure of oleanolic acid.

**Figure 19:**
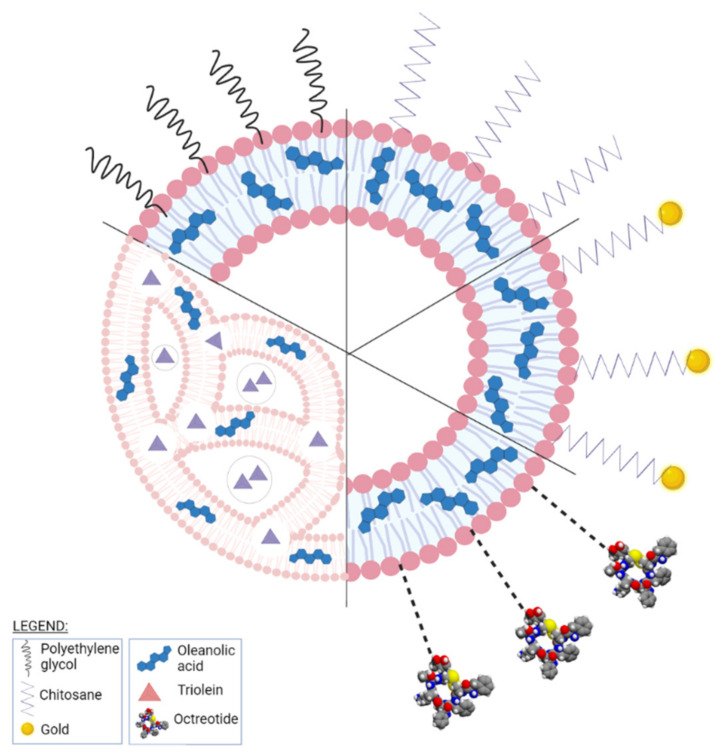
Schematic representation of different reported OA liposomal formulations. Created with BioRender.com (accessed on 10 January 2022).

Oleanolic acid (OA) is a pentacyclic triterpene (Figure 18) found in nature as a free acid or as the aglycone of triterpenoid saponins, regularly associated with its position isomer, ursolic acid [237,238]. OA can be extracted from edible and medicinal plants, reaching high concentrations in olive leaves (*Olea europaea* L.) [239]; it exhibits a broad spectrum of pharmacological activities such as antioxidant, anti-cancer, anti-inflammatory, cardioprotective, hepatoprotective, and anti-diabetic [240,241] and also shows a surprisingly significant inhibitory activity against HIV type 1 [242]. The various OA formulations discussed in this section are schematically represented in Figure 19.

Tang et al. carried out a study focused on increasing OA bioavailability by its inclusion in liposomes with modified surfaces; a modified ethanol injection method combined with sonication was used to encapsulate OA in PEG-ylated liposomes which provided good stability, solubility, and diffusion permeability for the active phytocompound, combined with a slow in vitro drug release which might lead to lower drug toxicity. The anti-cancer activity of these OA liposomes was tested in vitro against HeLa cells by MTT assay where cell viability was decreased in a dose-dependent manner in higher percentages compared to pure OA. The cytotoxic activity induced by the PEG-ylated liposomes displayed a similar pattern with pure OA; the presence of the hydrophilic PEG outer layer provided a significant reduction in the effective drug dose as well as a remarkable drug-loading ability (>98%) while also inducing a longer drug life by avoiding opsonization and macrophage uptake [243]. The researchers continued with the in vivo testing of the OA-loaded PEG-ylated liposomes on Kunming mice bearing U14 (cervical carcinoma) xenografted tumors; pure OA as well as OA-loaded liposomes were administered orally and significantly suppressed tumor growth with higher efficiency for the entrapped phytocompound by inducing tumor cell apoptosis. In addition, no signs of systemic toxicity occurred as reflected by the lack of pathological changes in renal or hepatic tissues [244].

Gold nanoshells-coated liposomes mediated by chitosan were developed by Luo et al. in order to entrap OA (GNOLs) for a combined approach as anti-cancer agents by using chemo- and photothermal therapy. The GNOLs were synthesized by the seed growth method using the chitosan’s amine groups to afford the liposome gold linkage. The formulations reached an average diameter of 172.03 nm and a suitable zeta potential for an easier accumulation in tumor cells. The highest reported encapsulation efficiency and stability related to OA was 77.52 ± 1.23%, when the liposomes were stored at 4 °C. The nanoformulation enabled the slow and controlled release of the active phytocompound depending on the pH value of the environment; the pH-responsive effect is induced by chitosan and provides GNOLs with a pH-mediated drug release in tumor tissues. Their anti-cancer activity was tested in vitro on 143B cell lines under NIR irradiation; the generated hyperthermia triggered drug release in a selective manner due to the possibility to control light properties in space and in real time. The GNOLs were assessed in vivo on U14 tumor-bearing mice by combining the effects of photothermal ablation and chemotherapy; the results showed a remarkable inhibition and apoptosis of tumor tissues [245].

OA has the ability to ameliorate organ toxicity of associated chemotherapeutics such as doxorubicin; Sarfraz et al. assessed organ toxicity for a liposomal combination of doxorubicin with OA used as treatment against hepatocellular carcinoma. Nine liposomal formulations were prepared at fixed ratios with their entrapment efficiency considered as the main criterion for selection as the most suitable experimental design; their particle size was situated between 85–200 nm for all formulations. The apoptosis assay was performed on HepG2 cells where the co-delivery of liposomal OA and doxorubicin showed a synergistic anti-cancer effect. The in vivo tests on HepG2 tumor-bearing BALB/c nude mice treated with the new liposomal formulation resulted in higher tumor growth inhibition compared to either OA or doxorubicin alone; moreover, the combination of doxorubicin and OA exhibited limited cardiotoxicity and lacked any histopathological changes in the main organs hence representing a promising future therapeutic strategy for hepatocellular carcinoma [246]. The same research group perfected the previously used ethanolic injection method by eliminating the extrusion process in order to improve the entrapment and release of both active compounds, single and combined, while preserving particle size. The anti-cancer activity of the resulting PEG-ylated liposomes was tested in vitro on HepG2 and KB cancer cell lines, respectively, by means of the MTT assay, and in vivo on tumor-bearing Kunming mice. The MTT assay showed a synergistic apoptotic effect of the two compounds against HepG2 cells when a fixed OA concentration was combined with various doxorubicin amounts or vice versa, either free or entrapped in liposomes; the synergism phenomenon produced the 50% reduction in the dose of one drug able to induce a 50% cell viability when used in association with the other. The in vivo tests revealed a longer half-life of the active drugs entrapped in the liposomal nanoformulation due to the presence of the PEG layer; the histopathological evaluation revealed the lack of toxic activity in liver, kidney, or heart tissues which was attributed to the antioxidant effect of OA that protects organs against oxidative stress. The authors concluded that the liposomal formulation of OA combined with doxorubicin not only decreased the effective dose of both compounds but also eliminated doxorubicin’s organ toxicity without diminishing its anti-cancer effect [247].

Multivesicular liposomes containing OA (OA-MVLs) were designed and developed as a treatment against hepatocellular carcinoma. OA-MVLs were prepared by the double emulsion method where the lipids (cholesterol, triolein, stearic acid) including OA and soybean lecithin are dissolved in a solvent after which are subsequently emulsified with two water solutions to obtain a water-in-oil-in-water double emulsion from which the solvent is removed by vacuum evaporation; the reported encapsulation efficiency for the OA-MVLs was rather high at 82.3 ± 0.61% while the in vitro release study revealed a drug release rate of 80.56 ± 1.27%/12 h; their anti-proliferative effect was tested on HepG2 cells using the MTT assay technique for different concentrations while in vivo tests were performed on murine H22 hepatoma-bearing mice. OA-MVLs inhibited the growth of human HepG2 cells and murine H22 hepatoma more efficiently compared to the pure phytocompound; in both in vitro and in vivo experiments, the liposomal nanoformulation released the active drug in a sustained manner thus leading to a longer circulation time for the entrapped phytocompound which prolonged the survival of the tumor-bearing mice. The histopathological evaluation showed no toxicity signs on the host; therefore, due to their simple preparation method combined with the promising biological effects, OA-MVLs were revealed as potential future candidates for the treatment of various types of cancer [248]. The same group continued their research by effectively trying to improve drug release by analyzing the variable factors such as lipid composition and process parameters; they employed response surface methodology, a collection of statistical and mathematical methods able to quantify the relationship between various controllable parameters and the triggered results, for the development of improved multivesicular nanoformulations in terms of particle size and encapsulation efficiency by modulating their components’ ratio. The optimized resulting formulation induced selective cell toxicity in vitro against two human hepatocellular carcinoma cell lines, SMMC-7721 and HepG2, thus showing potential to fight different cell types of hepatocellular carcinoma; cell viability percentages were much lower for the liposomal formulation compared to the pure phytocompound and the cytotoxic effect was dose dependent. Furthermore, even when used in low doses, OA-MVLs inhibited the adhesion, migration, and invasion of liver cancer cells without damaging normal liver cells [249]. Another research group involved polyvinylpyrrolidone (PVP) as a protective coating for OA-loaded liposomes by using the thin film dispersion-sonication method and reaching >90% drug-encapsulation efficiency; the resulting nanoformulations were orally administered to healthy adult male Sprague-Dawley rats in order to assess their in vivo pharmacokinetic parameters. Compared to commercially available OA, the liposomal OA exhibited approximately seven times the maximum plasma concentration of OA thus providing improved oral bioavailability [250].

Bian et al. developed lecithin-cholesterol-based liposomes encapsulating OA and coated with chitosan by using the ethanol injection method; the rationale behind using chitosan resides in its positive charge which is preferentially attracted to the negative charge of the tumor cells’ surface thus achieving targeted delivery to cancer cells. The chitosan-coated liposomes showed higher rigidity and stability compared to conventional OA liposomes thus preventing the premature release of the encapsulated drug. The authors reported high determined encapsulation efficiency of 94.7%. In addition, chitosan exerted an enhanced release of the active drug under acidic conditions (pH = 5.5), reaching a value of 97.74 ± 4.45% after 72 h (30% higher compared to the release at normal pH), thus proving suitable for tumor-targeted drug release. The cytotoxic activity of the chitosan-coated OA-loaded liposomes was tested in vitro using the MTT assay and revealed stronger effects compared to the control groups represented by pure OA as well as OA-loaded conventional liposomes. Therefore, the authors concluded that the chitosan-modified liposomal formulation loaded with OA might not only solve the low water solubility of the encapsulated drug but also provide an improved anti-cancer efficacy [251].

For the same purpose of achieving targeted delivery to cancer cells, Wang et al. developed octreotide-modified liposomes loaded with OA aimed to enhance the activity of the active drug against tumor cells overexpressing somatostatin; octreotide is an analog of the endogenous somatostatin which possesses a high affinity for the somatostatin receptors thus being able to direct the delivery of liposomes to the targeted cells. The octreotide OA liposomes (O-OA-L) were prepared by the ethanol injection method which produced an adequate particle size (100–200 nm). The O-OA-L exhibited a slow release of the encapsulated phytocompound (45.39%, after 72 h dialysis) and revealed that the presence of octreotide did not alter its release profile compared to conventional liposomes (46.27%, after 72 h dialysis). An increased inhibitory effect on the A549 cell line bearing overexpressed somatotropin receptors was recorded compared to free OA and OA-liposomes; no such increase occurred when the liposomes were tested against HeLa cells which show a low expression of the somatotropin receptors. Hence, O-OA-L exhibited high specificity for somatostatin receptors-overexpressing tumor cells thus providing the opportunity to achieve an optimized receptor-ligand affinity and, subsequently, an optimized targeting of the tumor cells [252].

#### 5.2.3. Glycyrrhetinic Acid

**Figure 20:**
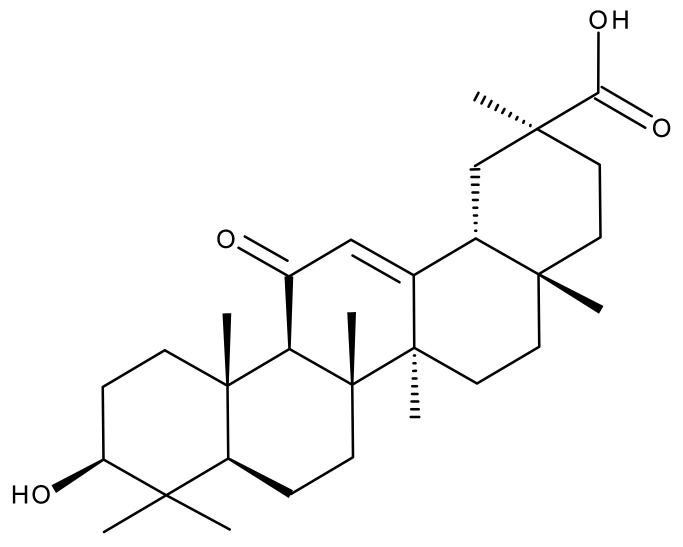
Chemical structure of glycyrrhetinic acid.

**Figure 21:**
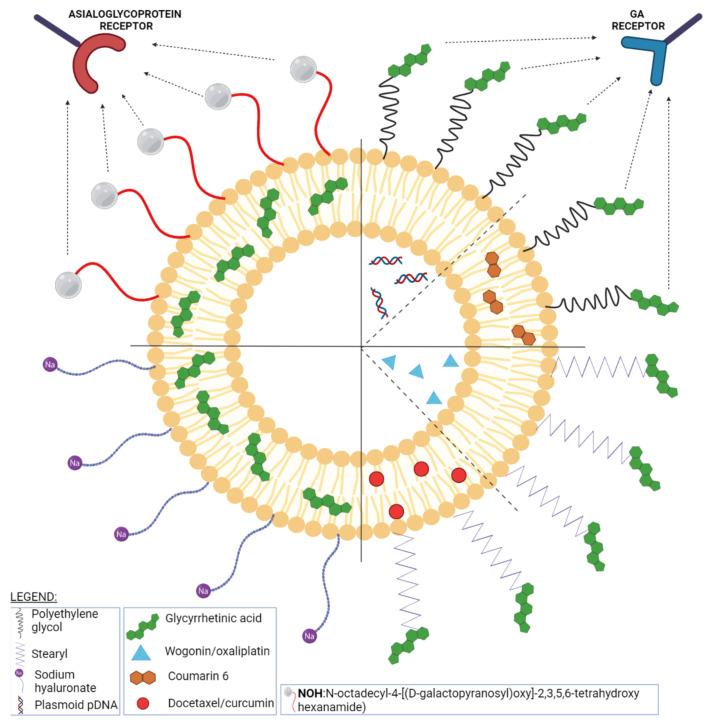
Schematic representation of different reported GA liposomal formulations. Created with BioRender.com (accessed 10 January 2022).

Glycyrrhetinic acid (GA) is a pentacyclic triterpenoid (Figure 20) serving as the active aglycone of glycyrrhizin, the main active component of licorice (*Glycyrrhiza glabra* L.) root extract [253]. GA is hydrolyzed during plant metabolism into two pentacyclic triterpenoids, 18α- and 18β-glycyrrhetinic acids, which are stereoisomers whose configurations exhibit different pharmacological profiles [254]. Furthermore, GA exhibits a wide range of biomedical activities, including antioxidant, antiviral, anti-diabetic, anti-ulcer, hepatoprotective, anti-microbial, neuroprotective, and anti-cancer effects [255,256]. The various GA formulations discussed in this section are schematically represented in Figure 21.

The cellular membrane of hepatocytes and hepatocellular carcinoma (HCC) cells possesses an abundance of GA receptors whose binding to GA or GA derivatives may depend on the ligand’s chemical structure. The two stereoisomers bearing C3-hydroxyl and C11-carbonyl active groups exhibit different stabilities, solubilities, and pharmacological effects including anti-proliferative and protective effects against drug-induced organ toxicity. The targeting effect of various GA configurations and groups was investigated by Sun et al. who designed PEG-ylated liposomes decorated with GA derivatives through amidation and assessed their activity in vitro, on HepG2 cells, and in vivo, on tumor-bearing BALB/c mice xenografted with mouse ascites hepatoma (H22) cells. The liposomes were obtained through the thin lipid film hydration method. The lipid film consisted of a 1:10 GA derivative: phospholipid molar ratio, cholesterol (20 mg), and coumarin 6 (2 mg). The GA-modified liposomes exhibited higher loading ability and stability compared to common PEG-ylated liposomes up to 82.40% encapsulation efficiency for 3-Ace-GA-Cou6-Lip as compared to 70.33% for conventional PEG liposomes. In addition, the presence of GA moieties induced a faster cellular uptake in vitro and a longer remanence in vivo in liver cancer cells while leaving the targeting abilities and the loading capacity unaltered. In terms of GA-active configurations, the authors concluded that the β-configuration hydrogen atom at the C_18_ position contributes the most to the targeting effect [257].

A similar study was conducted by Chen et al. who used GA as a targeting agent for the liver delivery of oxaliplatin. Oxaliplatin (OX) is a third-generation platinum-based anti-cancer drug that was used as a liposomal formulation in order to improve its activity against HCC. Surface modified oxaliplatin-loaded liposomes were designed by Chen et al. by adding GA to the lipid mixture during the thin film-dispersion method; in vitro testing indicated the slow release of the active drug compared to pure oxaliplatin. The GA-modified liposomes were tested in vivo on rats and Kunming mice where the pharmacokinetic studies revealed an improved absorption compared to conventional liposomes; biodistribution studies showed the preferential accumulation of oxaliplatin in liver tissue compared to the passive distribution to the heart, liver, lung, and kidneys that occurs for nonmodified liposomes thus certifying the efficiency of the GA molecule to act as a targeting agent for hepatocytes. In addition, no signs of organ toxicity such as epithelial necrosis were reported [258]. The hepatocyte-targeted delivery was also achieved for docetaxel-loaded liposomes that were surface modified with GA (GA-DDX-Lip). These soybean phospholipids-cholesterol-based liposomes were also obtained using the same method previously described, only this time using a different GA derivative, 3-succcinyl-18-stearyl GA (18-GA-Suc). The cellular uptake and cytotoxicity were tested in vitro on human hepatocytes cells L-02, nonparenchymal cells LX-2, human hepatocellular carcinoma SMMC-7721 cell line, and HepG2 cell line, respectively, and in vivo studies on BALB/c female nude mice xenografted with SMMC-7721 tumor cells. The biological assays revealed that the addition of GA acid to docetaxel-loaded liposomes enhanced their selective targeting to hepatocytes due to the receptor-mediated endocytosis. As a result, the modified GA-DDX-Lip displayed an improved anti-cancer activity both in vivo and in vitro compared to the unmodified liposomes, while retaining the same pharmacokinetic profile [259]. The research on docetaxel-loaded liposomes decorated with GA went further by co-loading copper sulfide nanoparticles which rendered the nanoformulation thermosensitive; thus, upon injection in liver tumor-bearing mice, the modified liposomes increased drug accumulation at the tumor site and actively targeted liver cells while the copper sulfide enhanced the release of the active compound after being NIR irradiated. The modified liposomal formulation can be used for photothermal therapy combined with chemotherapy thus exhibiting a significantly improved inhibitory activity of tumor growth as well as lower toxicity [260]. A similar study used GA as liver targeting ligand for wogonin-loaded liposomes (GA-WG-Lip); the liposomes were prepared by the reverse evaporation method using the same 18-GA-Suc derivative, only this time the active drug, being water soluble, was emulsified in the lipid solution, after which the solvent was removed through vacuum evaporation. The anti-cancer efficiency of GA-WG-Lip, pure wogonin (WG), and WG-loaded passively targeted liposomes was tested in vitro on HepG2 cells and in vivo on liver tumor-bearing mice. The results showed that the entrapment efficiency was not altered by the addition of GA which in turn induced higher cellular uptake (1.6 times higher) and similar IC50 value compared to conventional liposomes. A much-improved anti-neoplastic effect was recorded for GA-modified liposomes loaded with wogonin which were able to specifically target the liver with a long retention time and reduce tumor growth. Collectively, the experimental results indicated an optimized biodistribution, tumor accumulation, and anti-cancer efficacy for the GA-modified liposomes presumably due to the receptor-mediated cellular uptake of the nanoformulation [261]. GA also induced the liver targeted delivery of curcumin loaded in cationic liposomes; a complex of GA and octadecylamine was prepared through a simple static binding reaction and later used for the preparation of GA-modified curcumin-loaded cationic liposomes by means of the ethanol injection method. This formulation was highly suitable for curcumin entrapment given by the high entrapment efficiency value of 98.26 ± 1.33%. In vitro, the GA-modified liposomes produced a stronger inhibition of the HepG2 cells than free curcumin; however, the most remarkable results were revealed in vivo on H22 tumor-bearing mice where the anti-tumor effect following intravenous administration was similar to the one recorded for the intratumor administration. The in vivo experiment emphasized the inhibition of tumor growth, an anti-angiogenic effect, and improved hematologic parameters for the treated mice thus indicating a future potential treatment for liver cancer [262]. A further improvement of the hepatocyte-targeted delivery of an active drug was achieved by using surface galactose-modified liposomes which benefit from the recognition by the asialoglycoprotein (ASGP) receptors displayed at the hepatocytes’ surfaces. GA was used as a model drug to be loaded in liposomes whose surfaces were subjected to an enzymatic modification in order to include β-galactose residues. The in vitro assessment of the resulting liposomes revealed stable nanoformulations with high encapsulation efficiency (>90%) and sustained drug release [263].

A novel formulation of GA-modified cationic stealth liposomes (GA-PEG-CLs) was developed by He et al. in order to facilitate gene delivery to healthy and cancerous liver cells. The liposomes were obtained through the film dispersion method by mixing cholesterol, 1,2-dioleoyl-3- trimethylammonium-propane (DOTAP) a GA-PEG-cholesterol conjugate. Blank liposomes were incubated afterwards with a 5% glucose protein plasmid DNA solution. These formulations were subsequently tested in two different concentrations (1% and 5%) by comparison to cationic liposomes (CLs) and PEGylated cationic liposomes (PEG-CLs), respectively. The in vitro cell transfection efficiency tests on HCC HepG2 cells showed that the best transfection efficacy occurred for GA-PEG-CLs 5% which also displayed the lowest toxicity on normal L02 liver cells; for the human embryonic kidney cell line HEK 293 which does not overexpress the GA receptor, GA-PEG-CLs revealed the lowest transfection rate compared to CLs and PEG-CLs. Therefore, GA-PEG-CLs may serve as suitable, low-toxic gene carriers for hepatoma gene therapy [264].

The bioavailability and anti-cancer activity of GA can be improved by its co-encapsulation with salvianolic acid B and Tanshinone II A. The liposomes (GTS-lip) were prepared by the film hydration method combined with probe sonication which achieved the encapsulation of the two hydrophobic compounds, tanshinone IIA and GA, followed by the pH gradient method able to load the hydrophilic salvianolic acid B. Spherical liposomes were obtained with decreased in vitro release rate compared to pure drugs as well as clear and prolonged inhibitory effects against hepatic stellate cells. Therefore, due to the synergistically enhanced anti-proliferative activity against hepatic stellate cells combined with their sustained release effect, GTS-lip might serve as a potential future promising therapy in liver cancer [265]. The methoxy-PEG-poly(lactide) surface modification of GA-loaded liposomes provided the active drug with increased stability and encapsulation efficiency but, more importantly, it increased the half-life of GA thus improving its pharmacological profile [266].

GA liposomes not only display anti-tumor effects but may also protect the host against the organ toxicity of associated chemotherapy; Ge et al. explored the premise that GA-loaded liposomes could inhibit inflammatory stress and act as cytoprotective agents against nonbacterial cystitis induced by treatment with cyclophosphamide. Mature female Kunming mice were orally administered cyclophosphamide and were later tested for their blood levels of lactate dehydrogenase and cytokines (IL-6, TNF-α) as mediators that are overexpressed in injuries and inflammation. Treatment with GA liposomes induced significantly reduced blood levels of inflammation mediators; these results were endorsed by the histopathological and immunohistochemical analysis of bladder tissue which showed reduced inflammatory infiltration and cell death as well as the dose-dependent down-regulation of NF-κB and TNF-α expressions in treated mice thus certifying that GA liposomes might provide beneficial effects against drug-induced cystitis [267].

In addition to its use as an anti-cancer agent, GA can be used in cosmetics due to its nontoxic, anti-allergic, skin immunoregulatory, and whitening properties; formulations specifically suitable for cosmetic applications were developed by Jia et al. by preparing PEG-modified liposomes using a solvent-ultrasonic method and the PEG-7 glyceryl cocoate as GA solubilizer and penetration enhancer. Compared to the non-modified GA liposomes, the PEG-modified GA liposomes exhibited reduced particle size, higher zeta potential values, improved stability, and encapsulation efficiency as well as an efficiently controlled release; moreover, they managed to deposit a suitable amount of drug at the epidermal level thus allowing an efficient activity on the skin [268].

Nanosystems with GA also show benefits in wound-care; GA-loaded liposomes and hyalurosomes, respectively, were impregnated in various dressings and tested as alternatives to traditional cotton dressings. Hyalurosomes differ from liposomes through the presence of sodium hyaluronate which was selected due to its established role in wound healing. GA-loaded liposomes and hyaluorosomes were prepared using the film hydration method; their encapsulation efficiency exhibited similar values (~60%). In vitro studies showed that the release of the active drug from the nanosystems occurred within 30 min which indicates the fast achievement of maximum drug amount at the wound site. The release of GA from the final dressing was slower in the first 30′ presumably due to the dispersion of the nanosystems into the dressing environment; however, the complete release of GA was achieved within one hour. Taking into consideration that the first hour is essential in the wound healing process, the authors concluded that GA’s fast release may accelerate tissue repair. The biocompatibility of GA-loaded liposomes and hyaluorosomes, respectively, was determined in vitro on 3T3 fibroblasts using the MTT assay method; both nanosystems proved nontoxic but hyaluorosomes showed adequate stability and a stimulating effect on fibroblast proliferation thus significantly contributing to wound healing [269].

Conventional liposome preparation methods lead to aqueous suspensions which display poor long-term stability; an effective way to overcome this drawback is the preparation of proliposomes in the form of powders obtained through the dehydration of liposome components which may be dispersed in water before application thus regenerating the liposomes. Liu et al. developed such GA-loaded proliposomes using the lyophilization monophase solution method; the authors optimized the formulation and processing variables of GA liposomes in order to achieve adequate stability, encapsulation efficacy, and sustained drug release. In addition, the uptake process of regenerated liposomes by Hep G2 liver cancer cells was time dependent [270].

#### 5.2.4. Ursolic Acid

**Figure 22:**
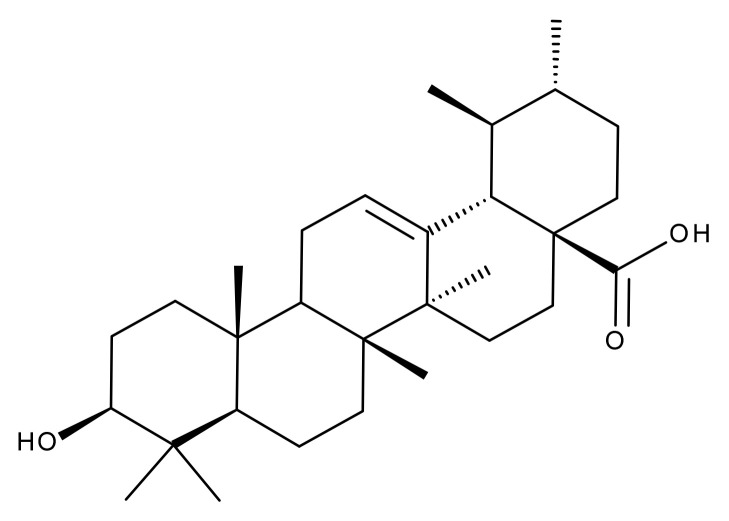
Chemical structure of ursolic acid.

**Figure 23:**
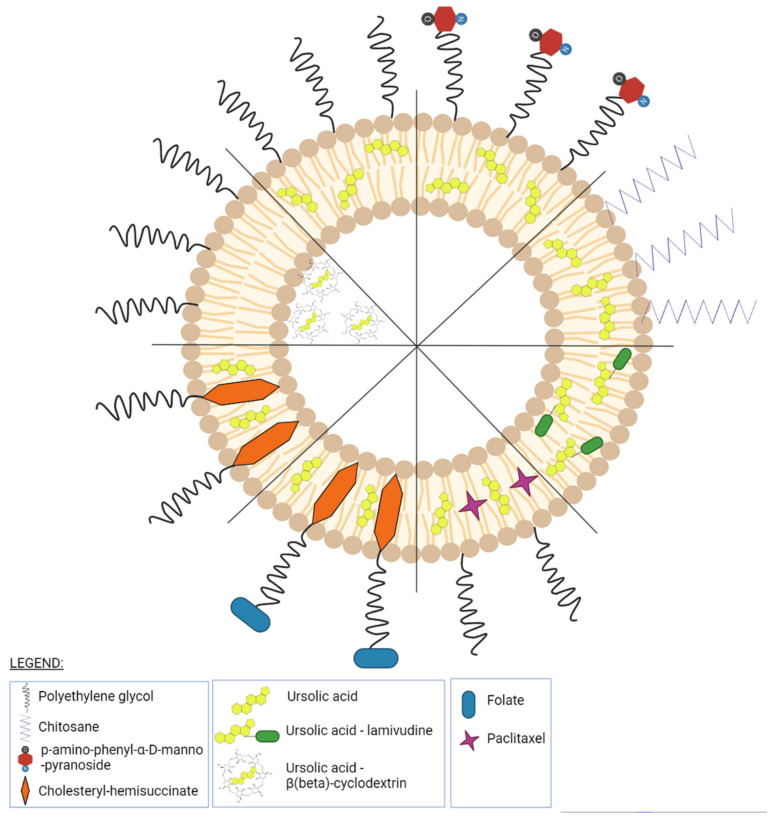
Schematic representation of different reported UA liposomal formulations. Created with BioRender.com (accessed on 10 January 2022).

Ursolic acid (UA) is a pentacyclic triterpene (Figure 22) abundantly found in nature in various fruits and vegetables but also dietary fibers and brown mustard as well as the leaves and herbs of the *Lamiaceae* family [271]; it was revealed as non-toxic and accumulates predominantly in spleen and hepatic cells [272]. Even though UA possesses a broad spectrum of impressive biological activities such as anti-allergic, anti-tumor, cardioprotective, analgesic, neuroprotective, anti-obesity, anti-anxiety, and anti-depression [273], its low bioavailability induced by its high lipophilic structure requires various chemical modifications in order to improve its potential pharmacological use [274]. The various UA formulations discussed in this section are schematically represented in Figure 23.

Due to its low water solubility and bioavailability, UA was incorporated in PEG-liposomes in order to modulate the tumor microenvironment and regulate T cell activity in cancer immunotherapy. The UA liposomes were prepared by the thin-film hydration method using a hydrophilic cyclodextrin (hydroxypropyl-β-cyclodextrin—HPβCD) as a solubility enhancer for UA, following a multiple-step process. UA was first transformed to a metformin salt after which this salt was used to form an HPβCD. This pre-formulation afforded an increased UA water solubility and therefore rendered the possibility of including UA in the internal cavity of the liposome as opposed to normal UA liposome formulations where the triterpene would be immersed in the lipid bilayer of the particle. This process enabled the formation of thermodynamically stable nanosized crystalline UA inside the liposomes which facilitate the sustained in vivo UA release (>40% drugs quantity remains inside liposomes after 72 h). The in vitro cytotoxicity studies were conducted on several cancer cell lines including the 4T1 murine triple-negative breast cancer and failed to reveal cytotoxic effects for the UA liposomes. However, when administered in vivo to tumor-bearing C57BL/6 mice, they were able to modulate CD4 + CD25 + Foxp3+ T cells thus correcting the tumor-mediated immune-suppressive microenvironment. Consequently, the tumor tissue may become more sensitive towards anti-tumor immune cells; therefore, the authors concluded that the UA liposome treatment was able to inhibit tumor growth and act as an immunotherapy agent in future anti-cancer therapies [275].

Modified liposomes coated with chitosan were developed in order to achieve the pH selective release of the drug load as well as an efficient accumulation in tumor tissues. The liposomes containing cholesterol, soy phosphatidylcholine, and UA were obtained using the ethanol injection method, after which chitosan (CS) was attached to the liposomal surface CS, a natural polysaccharide, was chosen as a liposomal modifier agent due to its propriety to open the tight junctions of the epithelial cells thus forming a paracellular pathway in the epithelial tissue. Furthermore, it augmented tumor targeting by increasing the positive charges on the liposome surface and as such, they tend to combine with the negative charges found on the surface of the tumor cells; therefore, the drug load (up to 94.3%) would be released at a pH of 5.0 compared to a physiological pH of 7.4. This pH-responsive drug release can presumably be attributed to the protonation of the amino groups of the chitosan chains in an acidic environment which causes the swelling of liposomes followed by UA release. The CS-UA liposomes also exhibited higher stability compared with conventional liposomes due to the electrostatic rejection between similar charges as well as slow and controlled drug release kinetics. The anti-cancer efficacy of the chitosan liposomes was tested in vitro on HeLa cells and in vivo on mice xenografted with U14 cells and revealed strong anti-proliferative effects with enhanced in vivo cell apoptosis and necrosis. The CS modifications of the UA liposomes proved to enhance their anti-neoplastic potential [276].

Another type of pH-sensitive long-circulating liposome containing UA was developed as an anti-angiogenic agent by using cholesteryl hemisuccinate which can be protonated in an acidic environment such as tumor cells, subsequently destabilizing the liposomes and releasing the active phytocompound at the tumor site. Due to the supplementary PEG chains, the newly developed nanoformulations possess long-circulating features able to protect them against degradation by the phagocytic system. The evaluation of their anti-angiogenic effect was conducted in vivo on murine brain cancer (9L cell line) and human breast tumor (MCF-7 cell line) models on nude mice. The results indicated poor anti-angiogenic effect against murine gliosarcoma (9L cell line) but demonstrated promising results against human breast adenocarcinoma (MCF-7 cell line); surprisingly, the inhibition of tumor growth was not reported in human breast tumor-bearing animals while a slight tumor inhibition was recorded for murine gliosarcoma-bearing animal [277].

Co-loaded PEG-ylated multifunctional liposomes were prepared by Ying et al. who used UA and epigallocatechin-3-gallate (EGCG) as apoptotic agents against C6 glioma stem cells. the liposomes were prepared using the film hydration method by using egg phosphatidylcholine, cholesterol and NH_2_-PEG_2000_-DSPE as lipid components; the liposome was later decorated with p-amino-phenyl-α-D-manno-pyranoside (attached to the end amino group of PEG using glutaraldehyde as linker) which may facilitate penetration through the blood–brain barrier and increase the targeting of glioma cells. Reported encapsulation efficiency for UA and EGCG was 78.90 ± 0.57% and 75.53 ± 0.56%. The biological activity was tested in vitro on C6 glioma cells and C6 glioma stem cells revealing that the combination of UA and EGCG strongly inhibited the proliferation of C6 glioma cells and C6 glioma stem cells in a synergic and additive manner, respectively due to increased apoptosis and endocytosis. When administered in vivo to C6 glioma cells–xenografted mice, the modified liposomes were able to prolong their survival and inhibit tumor growth in higher percentages compared to conventional liposomes due to the triple mechanism: Protection versus the endothelial system, increased delivery across the blood–brain barrier, and specific targeting to glioma cells [278].

The co-loading of UA was also experimented by Lv et al. who combined the phytocompound with paclitaxel and loaded the mixture in stealth liposomes (UA-PTX-Lip) as potential treatment for head and neck squamous cell carcinoma. UA-PTX-Lip were prepared using the thin-film dispersion hydration method using hydrogenated soy phosphatidylcholine, cholesterol, and DSPE-PEG_2000_ as lipid components; their anti-tumor activity was compared with paclitaxel liposomes (PTX-Lip) in vitro against HCS-3 cancer cells lines; the results showed that UA-PTX-Lip exhibited highly improved cytotoxicity on HCS-3 cells compared to PTX-Lip due to the presence of UA which exerts an increased oxidative effect on HSC-3 cells thus leading to an optimized apoptotic effect [279]. The combination drug obtained by linking UA and lamivudine (LMX) was prepared and evaluated for its anti-hepatitis B and hepatoprotective activity. The LMX liposomes were prepared by the thin-film hydration method coupled with sonication, using multiple lipid ratios in order to obtain the highest drug loading capacity and encapsulation efficiency (12.59 ± 0.19% and 91.28 ± 3.25%, respectively); in vitro tests showed the sustained release profile of LMX following an initial mild burst. The pharmacokinetic studies were performed on rats and were consistent with the in vitro studies, revealing higher gastrointestinal absorption, prolonged-circulation time, and an increased bioavailability after intravenous administration compared to the LMX suspension [280].

In order to avoid some drawbacks of conventional liposomes and to reduce the clearance rate produced by the reticuloendothelial system, stealth PEG-modified UA liposomes were prepared by the ethanol injection method; the liposomal nanoformulation revealed a highly improved entrapment efficiency (up to 99.56%) and stability as well as prolonged release compared to conventional liposomes. The anti-tumor activity was tested in vitro against EC-304 cancer cells, using the MTT assay method; PEG-ylated liposomes showed a slightly lower cytotoxic activity compared to conventional liposomes presumably due to the PEG barrier which hampered UA release. However, they are expected to provide UA with a longer-circulating time in the bloodstream thus reaching a more efficient anti-tumor activity [281]. An in vivo study of PEGylated UA-loaded liposomes was conducted on CD-1 female mice inoculated with U14 cervical carcinoma mouse cells; the results demonstrated that the presence of the outer PEG shell extended the half-life and reduced the release rate of the incorporated drug while the rate of tumor apoptosis was heightened compared to conventional UA liposomes. The histopathological analysis showed no signs of toxicity on liver and kidney tissues thus recommending the nanoformulation for further development in anti-cancer treatment [282]. Improved stealth liposomes were designed and developed by coating their surfaces with folate-PEG-cholesteryl hemisuccinate; their pharmacokinetics and anti-tumor activity on folate receptor-positive human oral cancer KB cells were later assessed. The folate-PEG-ylated liposomes significantly enhanced UA bioavailability and solubility while preferentially targeting the folate receptor-positive cells thus providing a possible basis for future oral cancer treatment [283].

#### 5.2.5. Lupeol

**Figure 24:**
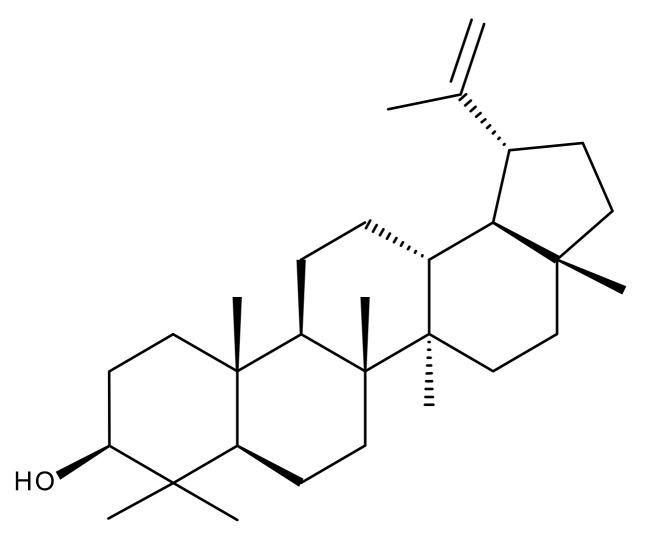
Chemical structure of lupeol.

**Figure 25:**
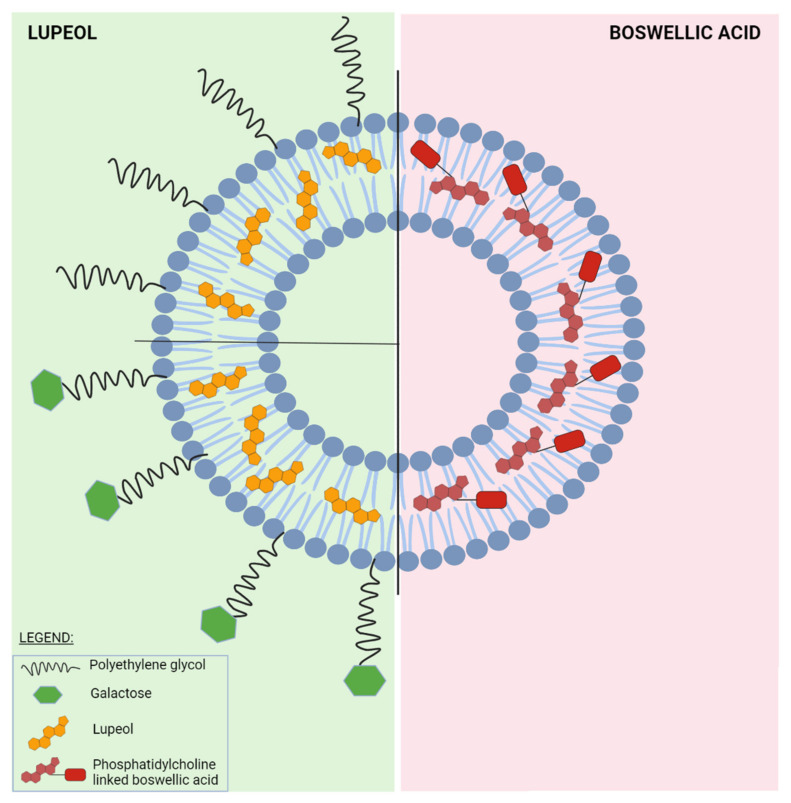
Schematic representation of different reported lupeol and BwA liposomal formulations. Created with BioRender.com (accessed on 10 January 2022).

Lupeol is a lupan-type pentacyclic triterpene (Figure 24) also found under the names clerodol, fagarasterol, and monogynol B; it can be extracted from a variety of vegetables and fruits such as soybean (*Glycine max* L.) Merr., aloe (*Aloe vera* L. Burm.f.), black tea (*Camellia sinensis var. assamica* J.W.Mast. Kitam.), tomato (*Solanum lycopersicum* L.), mango (*Mangifera indica* L.), etc. [284]. Lupeol exhibits a wide range of pharmacological proprieties such as cardioprotective, hepatoprotective, anti-diabetic, anti-cancer, anti-oxidant, anti-microbial, and anti-inflammatory [285]. However, its lipophilic structure induces low water solubility and bioavailability; therefore, requiring engineered formulations in order to exert an enhanced pharmacological activity [286,287]. The various lupeol formulations discussed in this section are schematically represented in Figure 25.

Lupeol-loaded liposomes bearing PEG chains were developed by Zhang et al. and tested in vitro on the HepG2 cancer cell line, revealing high inhibition and apoptosis rate, blocking the cells in the G2M phase; the PEGylation process improved the encapsulation proprieties (86.2 ± 0.9%) and zeta potential (1.6 ± 0.15 with PDI = 0.25 ± 0.029) thus contributing to an increased stability of the nanoformulation. In vivo studies were performed on rats by intravenous administration in order to investigate the pharmacokinetics of the liposomal formulation which increased the half-life and AUC of lupeol several times [288].

In a similar manner with GA, lupeol was incorporated in galactose-decorated liposomes in order to benefit from the asialoglycoprotein receptors found at the surface of hepatocytes and achieve a targeted delivery in liver cancer. Lupeol-loaded galactose-PEG-DSPE liposomes (GAL-L) were prepared by the thin-film dispersion method using HSPC, cholesterol, and galactose-PEG-DSPE as lipidic components; their encapsulated efficiency was above 85% and showed long-term stability. The apoptotic effect and cellular uptake were tested in vitro on the HepG2 cell line where GAL-L exhibited stronger effects and accumulation compared to the free drug or nontargeted liposomes. In vivo tests on wild-type FVB/N mice emphasized the liver-targeted delivery of GAL-L; as a result, the liver index as well as the liver weight of the respective treated mice displayed lower values compared to the non-targeted group. Histopathological analysis revealed that the GAL-L-treated mice had a clearer liver lobular structure with more obvious vacuoles and more abundant cytoplasm; in addition, liver cancer markers such as AFP, GPC3, and EpCAM mRNA exhibited lower expression levels compared to non-targeted lupeol-loaded liposomes [289].

#### 5.2.6. Boswellic Acid

**Figure 26:**
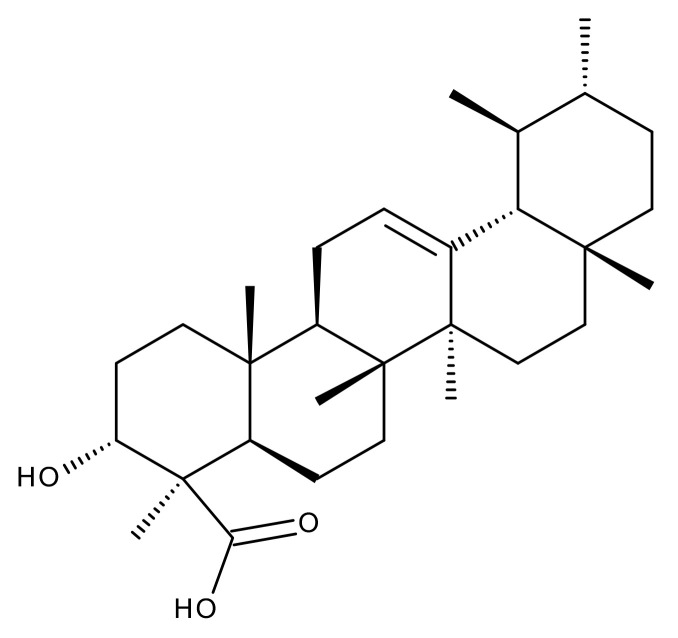
Chemical structure of boswellic acid.

Boswellic acid (BwA) is a pentacyclic triterpene (Figure 26) obtained from the gum resin of *Boswellia serrata* Roxb. ex Colebr., with a variety of applications in the fields of cosmetics, coating materials, and adhesives. More importantly, the phytocompound possesses a plethora of pharmacological activities such as anti-cancer, anti-septic, anxiolytic, analgesic, tranquilizing and anti-inflammatory [290,291]. Due to its highly lipophilic structure, its aqueous solubility and bioavailability are low, firmly requiring chemical or pharmaceutical modulations in order to allow oral administration [292]. The various BwA formulations discussed in this section are schematically represented in Figure 25.

Sharma et al. developed a modified phosphatidylcholine-boswellic acid complex (BA-PC) that was later encapsulated in three types of vesicular systems: liposomes, niosomes, and phytosomes. The BA–PC liposomes and phytosomes were prepared using the lipid film formation technique while niosomes were prepared by the reverse evaporation method; the BA–PC complex showed ex vivo increased absorption compared to pure boswellic acid in equimolar doses thus allowing a reduction in the drug’s dose and administration frequency. All the encapsulating nanocarriers were tested in vivo on rats for anti-inflammatory activity in carrageenan-induced paw edema and hypolipidemic activity in Triton-induced hyperlipidemia. The BA–PC complex showed an increased anti-inflammatory effect even compared to the phenylbutazone used as a reference and also induced stronger hypolipidemic effects as a result of increased absorption compared to the pure phytocompound. All three types of vesicular systems were revealed as more effective anti-inflammatory agents compared to phenylbutazone and boswellic acid, respectively, with phytosomes displaying the highest biological effects; this behavior is presumably caused by the small sizes of phytosomes which, combined with BA–PC complexation, greatly enhanced skin absorption [293].

## 6. Conclusions and Future Perspectives

The first marketed liposomal product was Doxil which contains doxorubicin as a loaded drug and was introduced in 1995 as an anti-cancer agent. Since then, research on liposomes has exploded, resulting in numerous new liposome types aimed for the treatment of various diseases. The use of liposomes provides researchers with the ability to adjust the pharmacological properties of the loaded drug—mainly its bioavailability and toxicological profile. The development of stimuli-sensitive and/or targeted liposomes has overcome the limitations of conventional therapies; as a result, several liposome formulations are already marketed or entered clinical trials, bearing anti-cancer agents, analgesics, vaccines, etc., including compounds of vegetal origin such as Vinca alkaloids, taxane, and campthotecine derivatives. Like many other classes of vegetal compounds, despite their numerous pharmacological properties and lack of systemic toxic effects, triterpenoids show one significant drawback represented by their low bioavailability presumably related to their lipophilicity. There have been many attempts to overcome this drawback such as the use of hydrophilic cyclodextrins, the formulation of nano/microemulsions, polymer carriers, and metallic nanoparticles; in all cases, the main challenges resided in the loading capacity, stability, toxicity, as well as the ability to overcome biological barriers. Each type of carrier exhibits its own disadvantage; for instance, non-biodegradable nanoparticles (e.g., gold nanoparticles) may induce toxic accumulation while nano/microemulsion may show inadequate long-term stability; moreover, some carriers come with high production costs which limit their accessibility. Liposomes have been revealed as efficient, non-toxic vesicular nanosystems able to incorporate the triterpenic scaffold by using relatively inexpensive materials and manufacturing procedures. In addition, liposomes showed a huge ability to support various modulations both at the surface and in the inner composition which promoted significant drug loading, sustained controlled drug release, improved pharmacokinetics, and targeted delivery thus inducing optimal pharmacological outcomes by avoiding acute and chronic organ toxicities as well as preventing premature drug degradation. Liposomes loaded with triterpenic compounds revealed higher pharmacodynamic effects compared to the drug alone, in particular as anti-cancer and anti-inflammatory agents, with limited toxicity on normal cells; moreover, triterpenes can be co-loaded with conventional anti-cancer drugs, managing to counteract their severe side effects. Besides serving as loaded drugs, triterpenes may also act as organ-targeting ligands as is the case of glycirrhetinic acid which is recognized by specific receptors located at the surface of hepatocytes and hepatocellular carcinoma cells. Liposomes are highly malleable carriers that are able to withstand various surface modulations; as such, chitosan-coated liposomes managed to achieve the pH-selective release of the drug load as well as an efficient accumulation in tumor tissues. In addition, chitosan was able to mediate the combination of liposomes with another type of nanocarrier, i.e., gold nanoshells, in order to provide the combination of chemo- and photothermal therapy. The long-term stability was improved by the preparation of proliposomes which may regenerate the liposomes as a result of water dispersion. One of the most important advantages liposomes can provide is their ability to bind various ligands used in targeted therapies; the targeted use of triterpenes has been proven highly useful in preclinical trials; however, with the exception of betulin and betulinic acid which have entered the pharmaceutical market or several clinical trials, respectively, triterpenes have not yet reached clinical expectations regardless of their pharmaceutical formulations. Through the advantages offered, their loading into liposomes may lead to sufficient improvement of their pharmacological profile so as to justify their inclusion in clinical trials; however, further research is mandatory in order to achieve this goal.

The fact that different types of liposome-based formulations are currently used in therapy or assessed in clinical trials stands as a driving milestone for the importance of developing such nanoformulations containing triterpenoid structures. There are, however, two important interconnected issues that need to be addressed. Firstly, the main triterpene representatives, especially the ones with anti-cancer activity, do not have a completely elucidated mechanism of action. Secondly, when available, the answers to this first issue should be coupled with a more accurate quantification of the liposome-released amount of substance at the tumor site, available to exert its pharmacological effect. After reaching these important checkpoints in the future, triterpenoid-containing liposomal formulations will be able to reach clinical trials and thus impact the current therapeutic landscape.

## Figures and Tables

**Table 1:** Types of nanocarriers used in imaging.

Category	Advantages	Drawbacks	Clinical Applications	References
Vesicle-type carriers (liposomes, micelles)	Increased potency and bioavailability	Instability Rapid clearance (traditional vehicles)	Drug delivery systems Imaging	[25,26,27,28]
Polymeric particles	Increased versatility Prolonged circulation time Improved stability	Morphology and size dependency Possible immunotoxicity	Drug delivery systems Imaging	[29,30,31]
Quantum dots	Unique optical proprieties Can identify numerous targets Various utilizations	Body toxicity produced by heavy metals Problematic efficiency High instability	Imaging Diseases molecular fingerprinting, Personalized diagnosis	[32,33,34,35,36]
Nanoshells	Significant reduced size Enhanced anti-tumor activity Sustained drug release proprieties	Are prone to aggregation Delicate stability balance	Imaging Drug delivery Photothermal therapy Tissue regenerations Gene screening	[37,38,39,40]
Gold particles	Easy to synthesize Great stability Non-immunogenic Hight surface area to volume ratio Great accumulation to the tumor site Possibility to conjugate a variety of moieties	High cost of synthesis Toxicity correlated to their size	Biomarkers Tumor labels Drug delivery systems Photothermal therapy	[41,42,43,44,45]
Paramagnetic particles	Greater magnetic susceptibility Better results in concentrating drugs in tumors Reduced treatment time	Cytotoxicity	Imaging Magnetic field targeting Diagnosis of pathologies	[46,47,48,49]
Carbon nanotubes	A broad area of clinical applications Great cellular permeability Impressive mechanical, electrical and thermal proprieties Natural affinity for diverse enzymes	Poor hydrosolubility Conductibility dependable on diameter and tube chirality Low biodegradability Toxicity	Imaging Drug delivery systems Gene therapy	[50,51,52,53]

**Table 2:** Classification of nanoparticles by routes of administration.

	Advantages	References	Drawbacks	References
Nanoparticles for intravenous drug delivery	- Drug steady state could be obtained faster and maintained easier - Fastest way to deliver an active compound into the bloodstream avoiding the difficulty of crossing the gastrointestinal mucosa	[55,56,57]	- Most dangerous route of administration - May cause irritation and tissue necrosis - Poor absorption by the tissues - Facilitates the risk for developing an addiction by using illicit drugs	[58,59,60]
Nanoparticles for oral drug delivery	- Easy administration - High compliance - Possibility of an easy self-administration - Flexibility in dosage adjustments and painless administration	[61,62,63]	- Low absorption rate due to the enzymes and bacterial flora present in the gut mucosa - Poor stability - Low bioavailability	[64,65,66]
Nanoparticles for transdermal drug delivery	- Reduced interfacial tensions - Prolonged delivery - Penetration through the skin of Both lipophilic and hydrophilic drugs - Avoiding the first-pass metabolism of the gastrointestinal tract	[67,68,69,70]	- Allergenic potential - Excessive local drug clearance - Only small lipophilic compounds can penetrate the skin	[71,72,73]
Nanoparticles for pulmonary drug delivery	- Higher safety - Little to no side effects - Delivery of big sized molecules and obtaining a steady distribution of the drug in the alveolar space	[74,75,76]	- The efficacy depends on many factors amongst which: the physicochemical proprieties of the drug and the condition of the patient	[77,78,79,80]
Nanoparticles in ocular drug delivery	- Intensifies the permeation and drug solubility thus providing a solution to the problematic means of classic administration of ocular treatment (poor bioavailability/low absorption in the ocular mucosa) - The toxicity and side-effects produced by the drug could be minimized	[81,82,83,84,85]	- Challenges in finding a suitable carrier for the drug that can enhance its proprieties	[86,87,88,89]

**Table 3:** Liposomal formulations used in therapy.

Brand Name	Therapeutic Agent	Route and Form of Administration	Indications	Status	References
Abelcet	Amphotericin B	i.v.	Fungal infections	Approved	[137]
Alocrest	Vinorebline	i.v.	Solid tumors	Investigational	[138]
AeroLEF	Fentanyl	Aerosol	Pain relief	Investigational	[139]
AmBisome	Amphotericin B	i.v.	Fungal infections	Approved	[140]
Amphocil	Amphotericin B	i.v.	Fungal infection	Approved	[141]
Aroplatin	Cisplatin	i.v./i.p.	Colorectal neoplasms	Investigational	[142]
Arikace	Amikacin	Aerosol	Cystic fibrosis	Investigational	[142]
Atragen	Tretinoin	i.v.	Solid tumors	Investigational	[143]
Atu027	siRNA	i.v.	Solid tumors	Investigational	[144]
Brakiva	Topotecan	i.v.	Solid tumors	Investigational	[145]
DepoDur	Morphine sulfate	Epidural	Pain management	Approved	[146]
DepoCyt	Cytarabine	i.v.	Lymphomatous meningitis	Approved	[147]
Dimericine	T4N4	Oral	Precancerous condition	Investigational	[148]
Doxisome	Doxorubicin	i.v.	Solid tumors	Investigational	[149]
Epaxal	Inactivated hepatitis A virus (strain RG-SB)	i.m.	Hepatitis A	Approved	[150]
Lipo-Dox	Doxorubicin	i.v.	Solid tumors	Approved	[151]
Lipoplatin	Cisplatin	i.v.	Solid tumors	Investigational	[152]
Liposomal alendronate	Alendronate	i.v.	Coronary artery stenosis	Investigational	[153]
Liprostin	Prostaglandin	i.v.	Peripheral vascular disease	Investigational	[154]
L-annamycin	Annamycin	i.v.	Acute lymphocytic	Investigational	[155]
Marqibo	Vincristine	i.v.	Solid tumors	Investigational	[156]
Mifamurtide	Mepact	i.v./injection/powder	High-grade, resectable, non-metastatic osteosarcoma in children and young adults	Approved	[157]
Nanocort	Prednisolone	i.v.	Rheumatoid arthritis	Investigational	[158]
NanoVNB	Vinorelbine	i.v.	Colon cancer	Investigational	[159]
Octinoxate	Eucerin, Meijer, Sumadan Wash	Topical/emulsion	Protection against UV light	Approved, Investigational	[160]
RVCLUV	Ropivacaine	i.v.	Anesthetic	Investigational	[161]
Stimuvax	BLP25 vaccine	i.v.	Lung cancer	Investigational	[162]
VaxiSome	Influenza	i.m.	Influenza	Investigational	[163]
7-ethyl-10-hydroxycamptothecin	-	*-*	Colorectal cancer	Investigational	[164]

**Table 4:** Liposomal formulations of various anti-cancer agents under clinical trials.

Drug Name	Therapeutic Agent	Indications	Phase Trial	References
BP1001	Grb2 antisense oligonucleotide	Leukemia, myelodysplastic syndrome, Ph1-positive CML	I	[165]
LiPlaCis	Cisplatin	Solid tumors	I/II	[166]
LDF01	Rhodamine-labeled cationic liposomes	Head and neck squamous cell carcinomas	I	[167]
Atu027	siRNA	Solid tumors	I	[168]
LEP-ETU	Paclitaxel	Ovarian cancer	II	[169]
OSI-211	Lurtotecan	Head and neck carcinomas	II	[170]
S-CKD602	TOPO I inhibitor	Solid tumors	II	[171]
MiR-122	MicroRNA	Hepatitis C	III	[172]
S9912	Paclitaxel, Cisplatin, Doxorubicin	Fallopian tube cancer, ovarian and peritoneal cavity cancer	II	[173]
ONYVIDETM	Ironotecan, 5-FU/LV	Advanced pancreatic cancer	I	[174]
Mitoxandrone	Mitoxandrone injection	Breast cancer	II	[175]
Abraxane combined with liposomal doxorubicin	Paclitaxel albumin-bound, Doxorubicin	Metastatic angiosarcoma	II	[176]
Nal-IRI + 5-FU/LV	Irinotecan + 5-fluorouracil/leucovorin	Pancreatic cancer	II	[177]
Paclitaxel	Camrelizumab + nedaplatin + apatinib + liposomal paclitaxel	Esophageal carcinoma	II	[178]
Amikacin liposome inhalation suspension	Amikacin	Mycobacterium avium complex lung disease	I	[179]
Pegylated Liposomal Doxorubicin	Trabedectin + Doxorubicin	Ovarian cancer	IV	[180]
Alprostadil injection	Alprostadil	Peripheral artery disease	II	[181]
LY01610	Ironotecan	Lung cancer	II	[182]
CPX-31	Cytarabine/daunorubicin	Acute myeloid leukemia	II	[183]

**Table 5:** The chemical structure of the most representative triterpenes.

Triterpene Types	Subtype	Chemical Structure
Lupane-type triterpenes	*Betulinic acid*	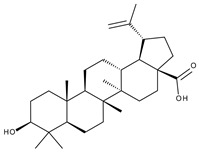
	*Betulin*	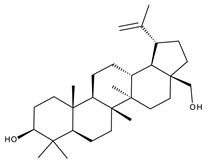
	*Lupeol*	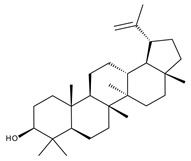
Ursane-type triterpenes	*Asiaticoside*	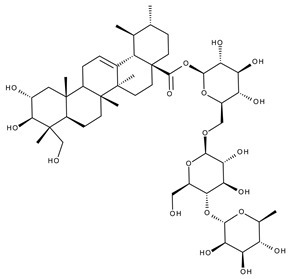
	*Asiatic acid*	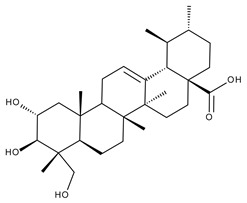
	*Madecassoside*	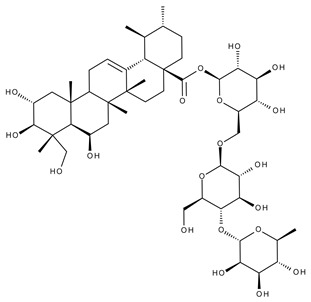
	*Madecassic acid*	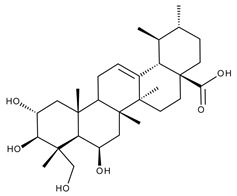
Oleane-type triterpenes	*Oleanolic acid*	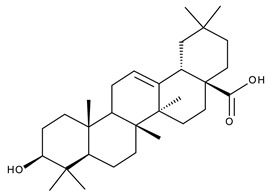
	*Glycyrrhizin*	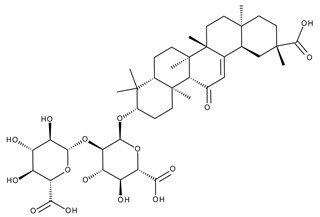
Dammarane-type triterpenoids	*Ginsenosides*	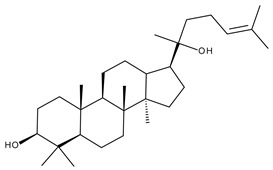
	*Bacosides*	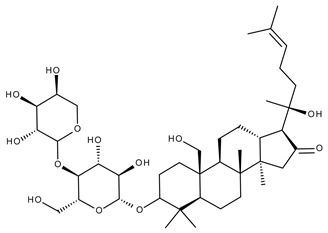
Lanostane-type triterpenes	*Cycloastragenol*	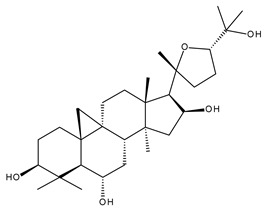
Cycloartane-type triterpenes	*Astragaloside*	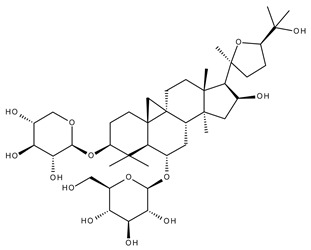
	*Cyclocanthoside*